# Joint analysis of mutational and transcriptional landscapes in human cancer reveals key perturbations during cancer evolution

**DOI:** 10.1186/s13059-024-03201-1

**Published:** 2024-03-08

**Authors:** Jae-Won Cho, Jingyi Cao, Martin Hemberg

**Affiliations:** The Gene Lay Institute of Immunology and Inflammation, Brigham and Women’s Hospital and Harvard Medical School, Boston, MA USA

**Keywords:** Cancer evolution, Clonal selection, Genetic-transcription perturbation, Metastasis, Drug resistance, Metabolism, Tumor microenvironment

## Abstract

**Background:**

Tumors are able to acquire new capabilities, including traits such as drug resistance and metastasis that are associated with unfavorable clinical outcomes. Single-cell technologies have made it possible to study both mutational and transcriptomic profiles, but as most studies have been conducted on model systems, little is known about cancer evolution in human patients. Hence, a better understanding of cancer evolution could have important implications for treatment strategies.

**Results:**

Here, we analyze cancer evolution and clonal selection by jointly considering mutational and transcriptomic profiles of single cells acquired from tumor biopsies from 49 lung cancer samples and 51 samples with chronic myeloid leukemia. Comparing the two profiles, we find that each clone is associated with a preferred transcriptional state. For metastasis and drug resistance, we find that the number of mutations affecting related genes increases as the clone evolves, while changes in gene expression profiles are limited. Surprisingly, we find that mutations affecting ligand-receptor interactions with the tumor microenvironment frequently emerge as clones acquire drug resistance.

**Conclusions:**

Our results show that lung cancer and chronic myeloid leukemia maintain a high clonal and transcriptional diversity, and we find little evidence in favor of clonal sweeps. This suggests that for these cancers selection based solely on growth rate is unlikely to be the dominating driving force during cancer evolution.

**Supplementary Information:**

The online version contains supplementary material available at 10.1186/s13059-024-03201-1.

## Background

Cancer is characterized by the accumulation of somatic mutations, resulting in distinct clones. The clonal composition of a tumor changes over time, and this evolution is one of the mechanisms by which new characteristics can be acquired during cancer progression [1–3]. Although evolution is gradual over short time scales, over longer times, more dramatic alterations may occur. New clones may induce clinically significant phenotypical changes or even physiological changes such as metastasis or drug resistance. These alterations are driven not only by mutations in protein coding regions that can change gene function but also by mutations in regulatory regions which can impact expression levels, thereby changing the range of phenotypes that a clone can attain [1–3]. However, our understanding of how the mutational and the transcriptional landscapes interact remains incomplete.

Single-cell technologies have enabled profiling both the genome and the transcriptome, providing important insights regarding tumor heterogeneity [4–7]. Even though scRNAseq does not profile DNA, one can infer both mutations and copy number changes from this data, making it possible to characterize both the mutational and the transcriptional landscapes. These technologies have been widely applied, including pancreatic cancer [8], acute myeloid leukemia [9], uveal melanoma [10], glioblastoma [11], and multiple cell lines [12], where they have provided insights regarding the interaction of mutational and transcriptional states. Another approach has been to combine single cell readouts with CRISPR technologies, allowing lineage tracing [13, 14]. In addition, associations of structural variation in the genome with cancer evolution have been reported [15, 16].

Even though the studies presented to date have provided important insights into cancer evolution, they face important shortcomings. Most studies use the 10X Genomics platform which only profiles one end of each gene, and it is thus only possible to characterize copy number variation [10, 17–21]. Some authors have incorporated genetic-transcriptomic perturbations to study cancer evolution, but only as low-throughput mutation profile [9] or using non-single-cell resolution [8, 22]. The limited resolution of these studies restricts our ability to investigate how mutations affect the transcriptional state at the single-cell level. Another shortcoming is that previous studies were either done in a mouse model [14], xenograft model [13], or cell line model [12], and consequently, they cannot fully account for the human tumor microenvironment (TME) which is known to play a key role. Also, those evolutionary studies are mainly focused on how primary cancer evolves rather than comparing between different contexts, such as metastasis or drug resistance, and the focus has been on histological classification rather than key phenotypical changes.

Here, we present Canvolution (https://github.com/jaewon-cho/canvolution/tree/master), a computational framework for analyzing cancer evolution and clonal selection from full-length scRNAseq data. Following mutation calling, the clonal hierarchy is inferred and along with the transcriptional profile this allows us to characterize the evolutionary paths. By focusing on gene signatures and pathways rather than individual genes, we identify broad trends across patients and cancer types [23]. In addition, we analyzed mutations affecting ligand-receptor interactions with the TME to infer its role in the evolution. By combining these analytical tools, we can compare the evolutionary trajectories of both the mutational and transcriptional landscapes between different contexts or perturbations in human cancer patients to characterize the sets of genes that are associated with changes between contexts.

## Results

### Canvolution compares mutation and transcriptome profiles from full-length scRNAseq

**Fig. 1:**
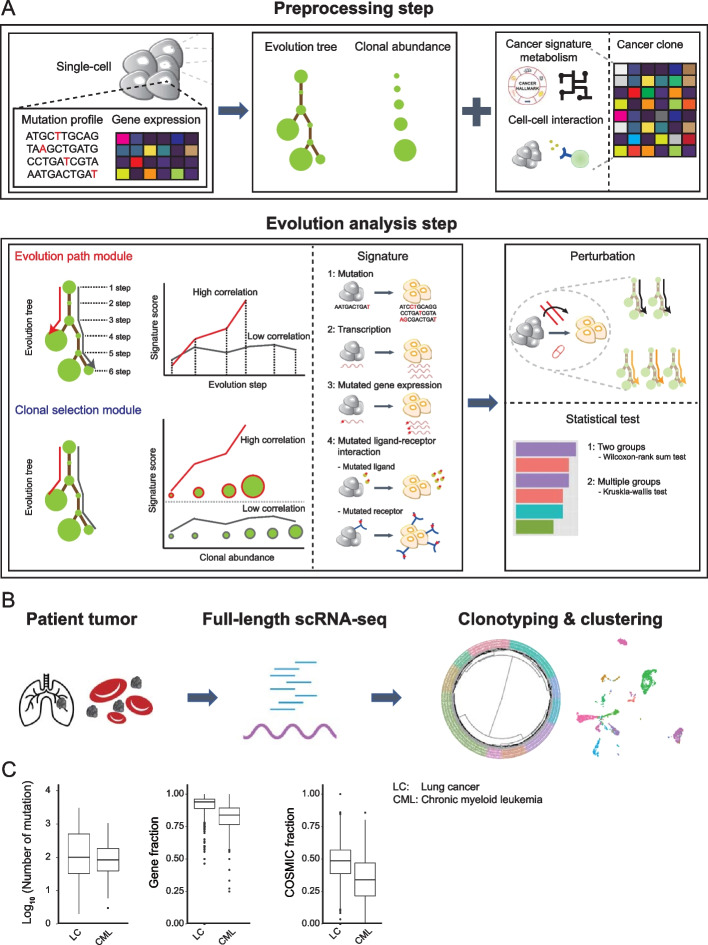
Overview and basic statistics of the data. **A** Schematic overview of Canvolution workflow. For the preprocessing part, mutation profiles and gene expression from single-cell RNAseq data are used as input. After generation of the evolutionary tree and calculation of the abundance of each clone, mutational signature scores and transcriptional signature scores are obtained by evaluating multiple cancer-associated signatures and metabolic pathways. With cell–cell interaction information, Canvolution can also generate a ligand receptor (LR) mutation score for each clone. The evolutionary path analysis measures the correlation between the signature score and the tree depth for each path. Similarly, the clonal abundance measures the correlation between the signature score and the size of the clone. **B** Schematic workflow of the research presented here. Tumor tissue from lung (LC) and chronic myeloid leukemia (CML) patients were used. **C** Boxplots showing the number of mutations (left panel), the fraction of mutated genes (middle panel), and the ratio of mutation that are assigned as COSMIC (right panel) per clone

Canvolution is a computational framework for joint characterization of the mutational and transcriptional landscapes; it consists of five steps (Fig. 1A): (i) preprocessing; (ii) identification of cancer clones and inference of evolutionary tree; (iii) characterization of clonal enrichment for each path through the tree; (iv) identification of transcriptional states through unsupervised clustering; (v) calculation of gene signature scores for mutation, transcription, mutated-gene expression, mutated ligand-receptor (LR) pairs in each clone-cluster combination. By default, single-nucleotide variants (SNVs) and short indels are identified using CTAT [24] in combination with a method based on the STAR aligner and GATK-best practice variant calling pipeline for inferring SNVs from full-length scRNAseq protocols [25]. This approach was chosen based on a wide review of the literature, including benchmarks to ensure robust performance [25]. Based on the mutations, clones are inferred using the DENDRO algorithm [7], and an evolutionary tree is generated by RobustClone [5]. Clustering of cancer cells by gene expression is done by standard Louvain clustering using the Seurat package [26].

To characterize clusters and clones, we use cancer hallmark gene sets from CancerSEA [27], cancer driver genes, oncogenes, and tumor-suppressor genes from CancerMine [28], as well as cancer fitness genes identified by a CRISPR-Cas9 screen across several cancer cell lines [29]. The latter collection includes core-fitness genes which were essential across 30 cell lines as well as cancer type-specific fitness genes. We also consider cancer testis genes (CTGs) [30], which are usually not expressed in normal tissue, but only in testis or tumor. Metabolic pathways are obtained from KEGG (hsa01100) [31], and cell–cell interactions are inferred using the CellChat package [32]. However, the framework is flexible, and instead of using the default settings, a user can input custom gene signatures or their own algorithms.

One of the main challenges when studying cancer evolution using patient data is that we typically only have access to a single snapshot of the tumor. Hence, we can only observe clones that have survived, and several assumptions are required to model the unobserved evolutionary paths. By comparing mutations, we can reconstruct the clonal tree and infer how clones are related, but we cannot determine if a clone is expanding or contracting. A key assumption is that samples were collected at a stage where all of the observed clones are expanding. By identifying features that are increasing or decreasing as a function of tree height, we can infer what features are associated with disease progression. In particular, we consider the mutations acquired along each path by defining a gene signature score, *M*_*s*,_ as the intersection between a set of pre-defined genes (e.g., ones associated with angiogenesis) with the mutated genes in a clone. Note that *M*_*s*_ is independent of changes in expression levels. We then calculate how *M*_*s*_ is related to the tree depth to determine if it is changing as the clone evolves, and we refer to the correlation coefficient between *M*_*s*_ and the tree depth as the evolutionary path score (Fig. 1A). Similarly, we can correlate the gene signature scores with the size of each clone, and this allows us to identify mutated gene sets that are associated with increasing clone sizes. We refer to the correlation between *M*_*s*_ and clone size as clonal selection score. To ensure that this approach is able to distinguish a true signal from noise, we first applied it to synthetic data (Methods, Additional file 1: Fig. S1). In addition, we can compare both evolutionary path scores and clonal selection scores between different conditions, e.g., primary vs metastasis, to identify statistically significant differences, i.e., mutated gene sets that are expanding over time in one condition but not the other. Similarly, we can carry out evolutionary path analysis and clonal selection analysis with transcriptional signature score, *T*_*s*_, which is defined by AddModuleScore in the Seurat package to observe transcriptional states associated with either clonal age or size. A special case of the transcriptional signature score is the mutated gene expression score, which is obtained by evaluating the transcriptional signature score by average expression level of mutated genes.

To evaluate changes in the interaction with the TME, we first infer interactions between the different malignant transcriptional clusters and the non-malignant cells. We then separate each of the malignant clusters based on their clonal identity, and we refer to this as a cluster/clone. For each cluster/clone, we calculate a mutated ligand-receptor (LR) score, *M*_*i*_, as the overlap between the mutated genes and the genes included in each interaction between other cell types. As before, *M*_*i*_ is calculated along each path of the clonal tree and correlated to either the depth or the size of the clone.

### Human cancers have degenerated mutational and transcriptional states

We analyzed two publicly available cancer datasets with two or more conditions (Fig. 1B, Additional file 2: Table S1), one solid cancer type (lung cancer, LC [17]) and one liquid cancer (chronic myeloid leukemia, CML [33]). The LC dataset includes primary and metastatic cancer as well as response to drug-treatment which is reported as progressive disease (PD), stable disease [34], partial response (PR), and complete response (CR). There were nine PD, two SD, 27 PR, and two CR; 28 of the samples were metastatic, and fourteen were from primary cancers. The LC dataset contained a total of 22,901 cells from 49 samples, with an average of ~ 3000 genes detected per cell. For CML, response before drug-treatment was given as poor or good. The CML dataset contained a total of 2224 cells from 51 samples, with an average of ~ 5500 genes detected per cell (Additional file 2: Table S1). Fifteen of the donors were good responders and fourteen had a poor response. For both LC and CML, information about the treatment response was not available for all samples, and some of the patients contributed more than one sample. Both datasets were generated by SMART-seq2, and following clonotyping, we characterized the mutational profile in each clone.

**Fig. 2:**
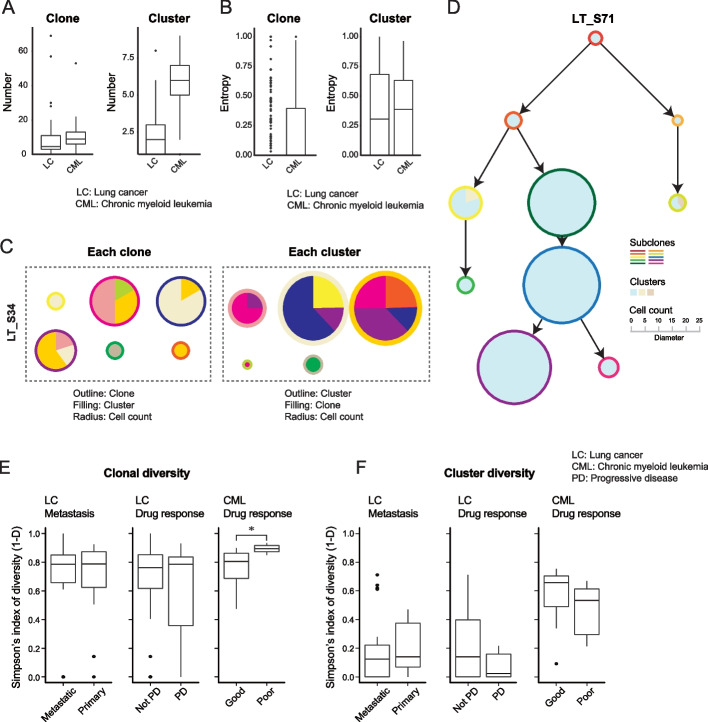
Preferable differentiation and degeneracy in the transcriptional state. **A** Boxplots showing the number of clones and clusters in each dataset. **B** Boxplots showing the entropy for the distribution of clusters in each clone (left) and the distribution of clones in each cluster (right). **C** One example of cluster heterogeneity in each clone from sample “LT_S34” in lung. The color of the outline of the circle indicates different clones. The diameter of the circle indicates the cell count of each clone. The colors of the pie slices indicate different clusters. The clonal heterogeneity in each cluster from the same sample is shown in the right panel. The color of the outline of the circle indicates different clusters. The diameter of the circle indicates the cell count of each cluster. The color of the pie indicates different clones. **D** One examples of an evolution tree from sample “LT_S71” in lung cancer. The color of outline of the circle indicates clone. The diameter indicates the cell count of each clone. The color of the pie slice indicates cluster. **E** Boxplots showing diversity for cancer clones between different contexts in each dataset. “*” indicates *p* value < 0.05 during the Wilcoxon-rank sum test (two-sided). **F** Boxplots showing diversity of cancer transcriptional clusters between the different contexts in each dataset. **E**, **F** “PD” indicates progressive disease samples, and “not PD” indicates samples without progressive disease (drug responders)

We first characterized the mutational landscape of the clones, and we found that the distribution of the number or mutations per clone showed large variability (coefficient of variation; LC: 1.478, CML: 1.051). As expected, most mutations were found in genic regions since the mutation calling was based on the expressed transcripts (Fig. 1C). The mutation burden, defined as the total number of mutations per clone, was higher in metastatic samples [35], but there was no difference based on the drug response status (Additional file 1: Fig. S2). On average, there were 16.9 mutations in driver genes, 76.1 in oncogenes, and 40 in tumor suppressor genes for the LC samples. The mutation profiles highlighted several well-known driver genes as 32% of samples had an EGFR mutation, 18% had a MET mutation, and 7% had a NF1 mutation [17, 36]. For CML, there were 2.4 oncogene mutations, 0.3 driver gene mutations, and 1.69 tumor suppressor mutations per sample, with 53% of sample containing a RUNX1 mutation and 38% a TP53 mutation [37] (Additional file 3: Table S2). Interestingly, among the mutated genes only ~ 50% were annotated in the COSMIC database [38], indicating a substantial number of mutations of unknown significance (Fig. 1C). The mean number of clones per patient was 9.66 for LC and 9.86 for CML (Fig. 2A), figures that are consistent with previous studies. For example, two separate studies using barcodes for lineage tracing, which can be considered the gold standard, report an average of 0.44 and 0.43 clones per cell [13, 39]. This is higher than what we found by a factor of 2.26 for LC and 2.88 for CML, but the discrepancy is not surprising as we are more likely to miss clones since we call mutations from the transcriptome.

We also considered the transcriptional landscape for each donor, and we found an average of 2.43 clusters for LC and 5.69 for CML (Fig. 2A). The smaller number of transcriptional clusters indicates a degeneracy with each transcriptional state consisting of multiple clones. We further confirmed the degeneracy by calculating the normalized entropy of transcriptional states (Fig. 2B, C), and this shows that observed entropies are lower than expected compared to a null model where mutational and transcriptional states are independent (Additional file 1: Fig. S3). This result reflects the fact that cells from the same transcriptional cluster are more likely to belong to the same clone, as indicated by the positive mutual information between the clone and cluster distributions (Additional file 1: Fig. S4). That is, there is a preferred transcriptional state for each clone, and conversely each clone has a preferable transcriptional state (Fig. 2B, C, and Additional file 1: Fig. S5).

### Evolutionary path analysis reveals gene sets associated with disease progression

Next, we considered the inferred tree structure for each donor, and a representative example of a clonal tree from a primary LC tumor from the partial response group is shown in Fig. 2D. We hypothesized that cancers with a more diverse clonal state are better at adapting to external perturbations since they are more likely to have a clone that has a high fitness in the new environment. Consistent with this hypothesis, we observe a significantly (*p*-value < 0.05, Wilcoxon test) higher diversity as defined by the Simpson index, for the CML samples from donors with a poor response to drug treatment (Fig. 2E). However, for LC, there was no significant increase of clonal diversity in metastasis or in patients with poor treatment response. Next, we asked if the diversity of transcriptional states is also associated with the ability to adapt to different conditions (Fig. 2F). There were no significant differences in transcriptional diversity between the different conditions, arguing against the hypothesis that higher fitness of a tumor is associated with a more diverse mix of transcriptional states. The transcriptional diversity is a global measure, and it carries little information regarding specific gene programs. Indeed, our analysis shows that in the LC dataset, genes related to metastasis (e.g., MET, RAC1, CD24) and epithelial to mesenchymal transition (e.g., FBLN2, SDC1, CTSB) were differentially expressed in metastasis samples. Similarly, in the CML data, we found that genes related to DNA damage (e.g., RBL2) were highly expressed in poor responders.

**Fig. 3:**
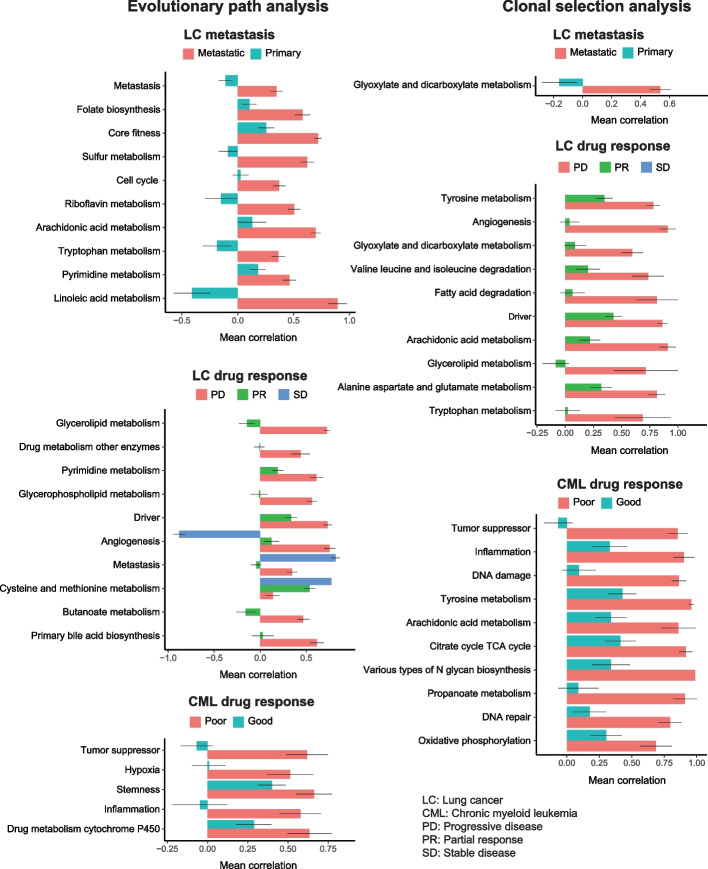
Mutational analysis during cancer evolution. Horizontal bar plots of evolution path analysis (left) and clonal selection analysis (right) with mutation features. “metastasis” versus “primary” (LC: Metastasis panel) and “progression disease (PD),” “partial response (PR),” and “stable disease” (SD) in lung cancer (LC: drug response panel). “good response” and “poor response” in CML sampled at diagnosis (CML: drug response panel). Only the top 10 terms with *q* value <  = 0.1 were shown. The bar indicates the mean Spearman correlation coefficient from each group. The standard error is shown as an error bar

As there is evidence in favor of clonal diversity being associated with higher cancer fitness, we investigated what features are selected for during cancer evolution. We compared the evolutionary path scores between different contexts to identify mutated gene signatures that are overrepresented in clones found at lower depths of the inferred trees. Reassuringly, the comparison between primary and metastatic tumors reveals that the most highly enriched category is metastasis (Fig. 3, Additional file 1: Fig. S6, Additional file 4: Table S3). This is consistent with previous reports of mutations in lung cancer fitness [40], and it reflects the higher incidence of mutations such as KEAP1, NFE2L2, EGFR, and MYC. In addition, we find an enrichment of mutations in driver genes, e.g., EGFR [41], KRAS [42], BRAF, and PIK3CA [43], from PD patients in LC. Similarly, we identify mutations in both tumor-suppressor genes, e.g., ABL1, JAK2, MAP2K1, and KIT [38], from poor responders in CML, suggesting that these are two of the key mechanisms by which tumor cells evolve to acquire drug resistance. We also compared the clonal abundance scores, and we find a similar set of gene signatures. To investigate if more advanced clones tend to be larger, we calculated the Spearman correlation between the clonal abundance and the height in the clonal tree, and this showed moderate relation (LC: 0.5, CML: 0.32). Hence, there is only modest evidence to support the hypothesis that more advanced clones have strong growth advantage over their predecessors.

### Transcriptional evolution analysis reveals preferable gene sets for each context

**Fig. 4:**
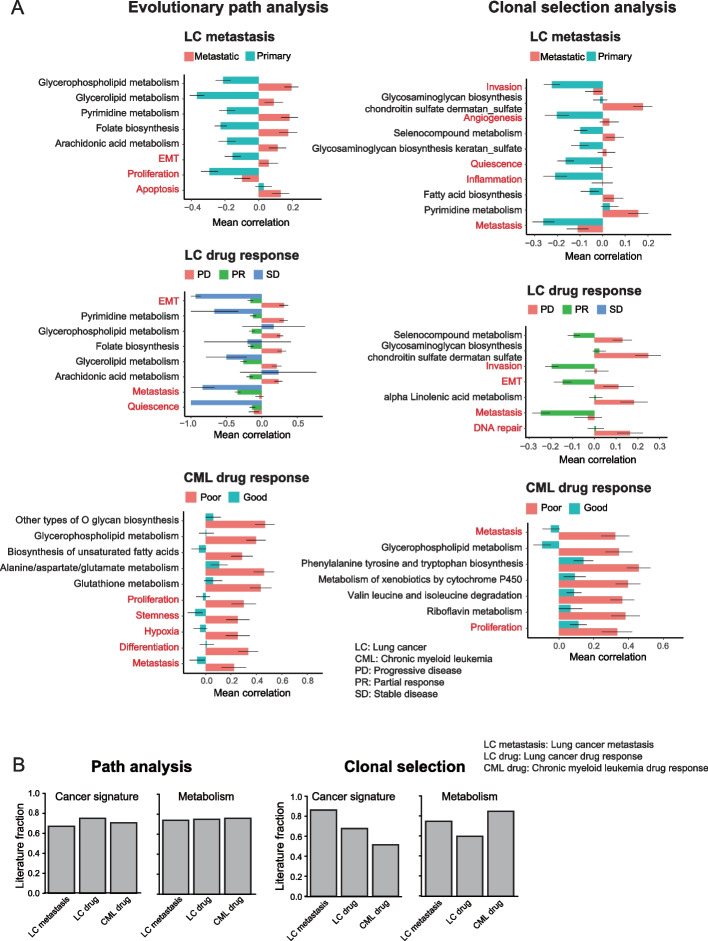
Transcriptomic analysis during cancer evolution. **A** The horizontal bar plots of evolution path analysis (left) and clonal selection analysis (right) with mutation features. “metastasis” versus “primary” (LC: Metastasis panel) and “progression disease (PD),” “partial response (PR),” and “stable disease” (SD) in lung cancer (LC: drug response panel). “good response” and “poor response” in CML sampled at diagnosis (CML: drug response panel). Only the top 5 cancer signatures and top 5 metabolisms for “metastasis,” “PD,” and “poor response” with *q* value <  = 0.1 were shown. All the results are shown in Fig. S6. The bar indicates the mean Spearman correlation coefficient from each group. The standard error is shown as an error bar. Terms in red are cancer related and terms in black are metabolism related. **B** Fraction of significant features from “metastasis,” “PD,” or “poor response” that were verified by literature search

Next, we explored transcriptomic perturbation during cancer evolution, and similar to the mutational analysis, we found that specific gene signatures were altered when comparing the different contexts (Fig. 4A, Additional file 1: Fig. S7, Additional file 4: Table S3). To validate these results, we carried out a literature search which revealed that the majority of the transcriptional changes that emerge in the metastatic or drug-resistant contexts have ample support (Fig. 4B, Additional file 5: Table S4, Additional file 6: Table S5). For example, in metastatic LC, we found enrichment for EMT [44], metastasis [27], differentiation [27], and several metabolic processes including pyruvate-lactate, amino acid (glutamine, serine, alanine, proline, asparagine), and lipids (fatty acid, acetate, acetyl-CoA) [45]. We also found known drug resistance features from LC (EMT [46], metastasis [47], quiescence [48], invasion [49], and angiogenesis [50]) and CML (DNA_repair [51], proliferation [27], stemness [52], hypoxia [53], and differentiation [54]), as well as metabolic processes involving pyruvate-lactate [55], amino-acid (glutamine, proline, serine, alanine, asparagine) [45, 56–58], lipids (fatty acid, acetate, acetyl-CoA) [59], and drug metabolism. Interestingly, our analysis highlights vitamin C (ascorbate) metabolism as enriched in metastasis or drug-resistant contexts. This essential nutrient was previously reported as an anti-metastatic or an anti-cancer agent [60, 61], and since vitamin C cannot be synthesized in human, we conjecture that this reflects catabolism to reduce its anti-tumor effect. Compared to the mutation analysis, the changes in transcriptional state for both evolutionary paths and clonal selection are more diverse with many more processes enriched across conditions (Figs. 3 and 4A, Additional file 1: Fig. S6 and S7).

### Mutational and transcriptional profiles are interlinked

**Fig. 5:**
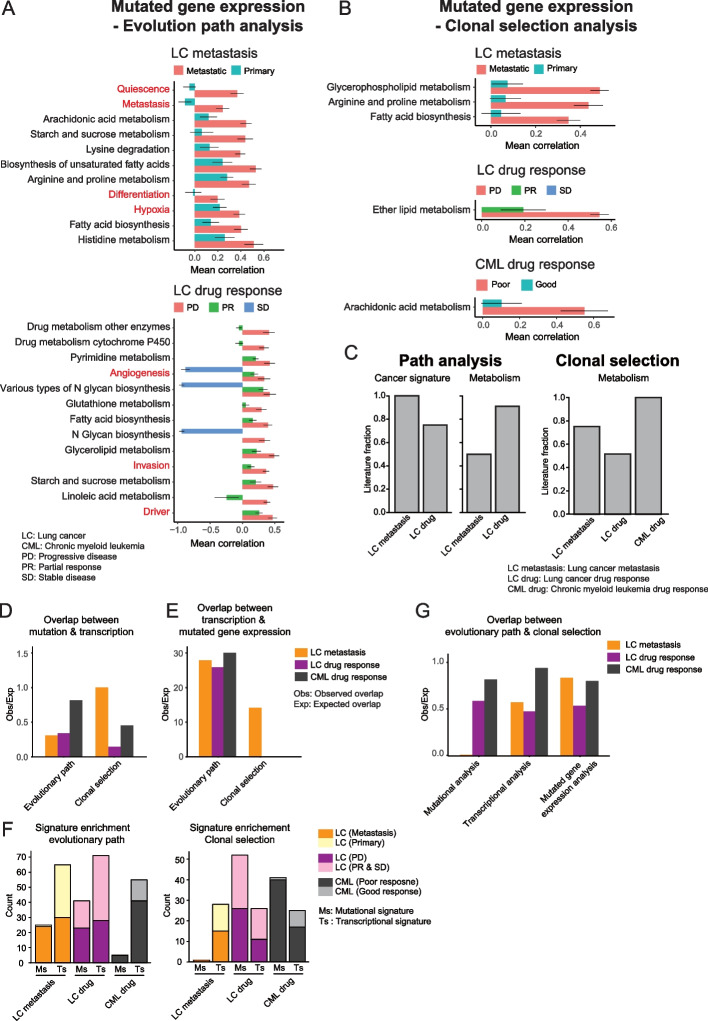
Integrated mutational and transcriptomic analysis reveals clonal selection and degenerate state in cancer evolution. **A**, **B** The horizontal bar plots of evolution path analysis (**A**) and clonal selection analysis (**B**) with mutated gene expression. “metastasis” versus “primary” (LC: Metastasis panel) and “progression disease (PD),” “partial response (PR),” and “stable disease” (SD) in lung cancer (LC: drug-response panel). “good response” and “poor response” in CML sampled at diagnosis (CML: drug-response panel). Only the top 5 cancer signatures and top 5 metabolisms for “metastasis,” “PD,” and “poor response” with *q* value <  = 0.1 were shown. The bar indicates the mean Spearman correlation coefficient for each group. The standard error is shown as an error bar. Terms in red are cancer related and black metabolism related. **C** Fraction of significant features from “metastasis,” “PD,” or “poor response” that was verified by literature search. **D** The overlap between significant features (*q* value <  = 0.1) in mutational analysis and transcriptional analysis in the evolutionary path and clonal selection analyses. **E** The overlap between significant features (*q* value <  = 0.1) in transcriptional analysis from the “metastasis” or “PD” group and mutated gene expression analysis in the evolutionary path and clonal selection analyses. **D**, **E** For the chemotherapy response analysis in lung cancer, we merged PR and SD. We considered the overlap if the feature is enriched in the same group. **F** Number of significant features of “metastasis,” “PD,” or “poor response” from Figure S6A and B for mutational analysis (upper panel) and Figure S7A and B for transcriptional analysis (lower panel). **G** Significant features overlap with the same enrichment group (mutational signature, transcriptional signature, and mutated gene expression; *q* value <  = 0.1) between the evolution path analysis and clonal selection analysis. **D**, **E**, **G** The bar plots indicate the observed overlap/expected overlap (Obs/Exp). Please see the detailed procedure in the “Methods” section

After exploring the evolution of the mutational and transcriptional landscapes separately, we analyzed them jointly. First, we asked if genes that are mutated also are more likely to have changes in their expression levels. When considering genes that are both mutated and have their expression levels perturbed, we found several gene signatures that were statistically significant in different contexts (Fig. 5A, B, and Additional file 1: Fig. S8). To validate these gene signatures, we carried out a literature search to determine if the expression of those signatures is associated with each context. Again, we found that the majority had been reported in the literature (Fig. 5C). Next, we tried to understand the coherence of the changes in the mutational and transcriptional profiles. When we compared the signatures that exhibited both mutations and altered expression levels, it was smaller than expected by chance for all three comparisons between evolutionary contexts (Fig. 5D). For example, even though patients from the LC cohort with PD showed an increased number of mutations in driver genes or angiogenesis mutations for both the evolutionary path and clonal selection analysis, these genes did not show increased expression levels. This result suggests that genes that are mutated do not also have changes in gene expression. By contrast, the metastasis or drug-resistant groups were more likely to express a mutated gene if those features were enriched in the transcriptional evolution analysis (Fig. 5E). This implies that for more advanced cancer, many of those pathways are expressing the mutated form, and if the mutation resulted in a gain or loss of function then the function may differ from the annotated one.

To understand the relative importance of changes in the mutational and transcriptional landscapes, we compared the number of significant features from the two landscapes. For both the path analysis and the clonal selection, we identified all mutational and transcriptional features that were enriched in each context. Comparing the two contexts, we found that the majority of the mutated gene sets were enriched in the more advanced stage, i.e., metastasis or drug resistance, compared to the less advanced stage. This finding is consistent with the notion that clones with many mutations in these genes have higher fitness and are better at adapting (Fig. 5F). By contrast, there was no such enrichment for the transcriptional states. We also compared the evolutionary path and the clonal selection analyses to identify shared trends between clones. Surprisingly, there was a high discordance between evolutionary path analysis and clonal selection with less overlap than expected by chance (Fig. 5G). Reassuringly, these conclusions are robust with regard to the choice of statistical threshold (Additional file 1: Fig. S9), and taken together, this suggests that maximizing growth rate is not the main force determining cancer evolution.

### Mutated ligand receptor shows how cancer evolution is influenced by the TME

**Fig. 6:**
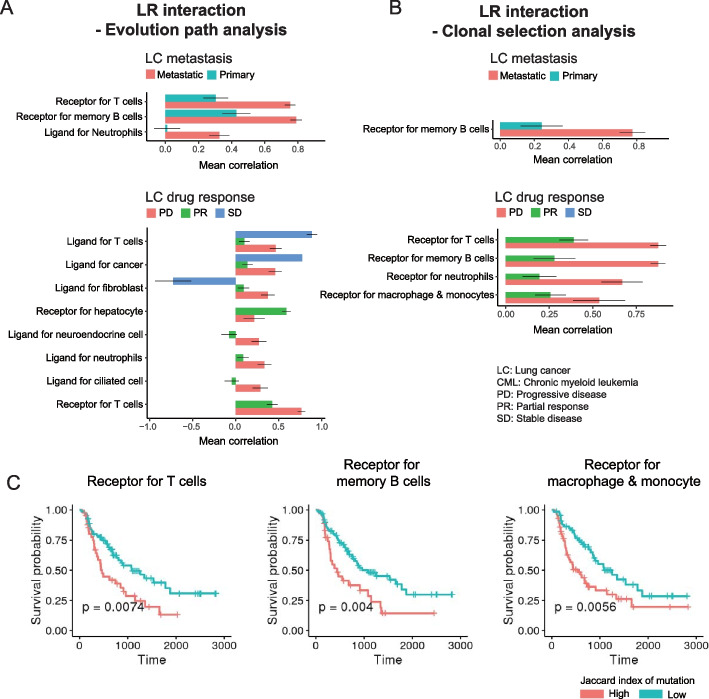
Perturbation of ligand/receptor reveals cooperative behavior during cancer evolution. **A** Results from evolutionary path analysis of mutated ligand-receptor interaction. Only the *q* value <  = 0.1 was shown. **B** Result from clonal selection analysis of mutated ligand-receptor interaction. “metastasis” versus “primary” (left panel) and “progression disease (PD),” “partial response (PR),” and “stable disease (SD)” in lung cancer (right panel). Only the *q* value <  = 0.1 was shown. **A**, **B** The bar indicates the mean spearman correlation coefficient from each group. The standard error is shown as an error bar. **C** Kaplan–Meier curve of non-small-cell lung cancer patients in TCGA data with each category. Jaccard index of mutation was obtained between mutation profiles in each sample with mutated receptors interacting with a given cell type

So far, our analyses have focused on cell intrinsic factors of cancer evolution. However, interactions between cancer cells and the TME are important [62, 63], and therefore, we investigated how cell–cell interactions between cancer cells and the TME are altered during cancer evolution in LC (Fig. 6A, B). The analysis is similar to before as we asked if either ligands (source) or receptors (target) relevant to the interactions with other cells in the TME showed enrichment of mutations. Considering both features that increase with lower depth (evolutionary path analysis) and features related to increased clonal abundance (clonal selection analysis), we identified mutational enrichment of ligands or receptors for cancer cells in different contexts, confirming that cancer evolution also impacts cell–cell interactions.

The clonal selection analysis revealed that only receptors on malignant cells were enriched. By contrast, four out of six trends with significant enrichment of mutations in ligands produced by cancer cells were found in drug-resistant tumors, suggesting that there is a benefit to malignant cells that alter the signaling molecules communicating with the TME. Interestingly, one of the top hits was ligands for interactions with cancer-associated fibroblasts (CAFs) (Fig. 6A). This result is consistent with the notion that cancer cells can interact with CAFs to reshape the TME to support their survival, and it is why CAFs are sometimes referred to as tumor promoting cells [62, 64].

The mutations in ligands and receptors suggest a role for cell-extrinsic effects during cancer evolution. Consequently, we hypothesized that the mutated ligands from cancer cells will impact CAFs to make them produce cancer-promoting ligands in the PD group, but not in patients where the disease is stable or reduced. Indeed, the ligands produced by CAFs in the PD group were enriched for the gene signature “cancer proliferation” (*p*-val: 1.67e − 3; Fisher’s exact test), while ligands specific to the non-PD group were not (*p*-val: 6.87e − 2). For example, both WNT5A [65] and FGF1 [66] were found in PD donors, but not in non-PD groups. In addition, we found an enrichment of mutations in receptors on cancer cells that reduce the impact of T cells and B cells (Fig. 6B). We also found mutations in receptors against macrophage/monocyte interactions, and cancer cells may strengthen this interaction since anti-inflammatory macrophages are known to promote cancer survival [17, 63, 67].

This result implies that patients with mutations in immune relevant receptors will have a worse outcome. To test this prediction, we analyzed receptors for interactions with T cells, B cells, macrophage, and monocytes in non-small cell lung cancer patients from The Cancer Genome Atlas. Splitting the cohort into patients with either a high or low number of mutations overlapping the receptors of interest, we found a significant difference in survival time (Fig. 6C, Additional file 7: Table S6). This finding yields clinically relevant information as it highlights genes associated with cancer progression. Many of these genes are well known, e.g., TNFRSF1A (TNFR1) or TGFBR2 which can be used against T cells to block apoptosis signaling [68, 69] or tumor suppressor signaling [70].

## Discussion

Both genetic and transcriptomic aspects are important for understanding cancer evolution. Together, they reveal the characteristics of cancer progression and how the disease adapts to external perturbations. However, most of the data from functional experiments comes from cell lines or from animal models. Here, we jointly explore the mutational and transcriptional landscapes at single cell resolution in human cancer patients. Understanding the evolution pressure in cancer started to get attention recently using lineage tracing technologies [13, 14]. Another method that we are aware of is the ASCETIC framework [71], which can use bulk and single-cell DNAseq data to infer a clonal tree and associated evolutionary signatures. However, ASCETIC is much more limited than Canvolution, as it can only infer mutation patterns that are related to patient survival. Thus, none of these methods can be applied to scRNAseq data from human cancer patients.

Our analysis showed a degeneracy in the transcriptional states, with more than one clone in each transcriptional cluster. Surprisingly, there was no strong evidence suggesting that more diverse tumors are associated with higher fitness, neither based on mutations nor transcriptome (Fig. 2E, F). Interestingly, CML samples show a higher clonal diversity for poor responders, while LC samples do not. We speculate that the difference could be due to CML being a liquid cancer while LC is a solid cancer. The two types of malignancies differ in terms of their interaction with the TME, and this may be reflected by the treatment response. Instead, we found that particular gene signatures were significantly enriched when comparing the evolution under the metastatic and drug-resistant conditions with the primary and drug responsive ones (Figs. 3 and 4A).

We find little evidence of clonal sweeps as all samples maintain a relatively high diversity of clones. This is unexpected since for a large enough population of tumor cells, newly evolved clones need to have increased fitness to explain the fact that they are able to survive among the existing ones [72]. We found additional evidence suggesting that viewing cells in isolation is insufficient. By contrast, we found that mutations frequently affect ligands or receptors, resulting in a change of interactions in the TME. Based on these findings, we conjecture that clones are not always competing against each other for survival. Instead, we found evidence suggesting that the tumor benefits from a diverse ensemble of clones in the drug-resistant model. This finding is consistent with previous reports about cooperation of cancer clones, but none of these studies considered the evolutionary implications or the association between the TME and cancer [73–75]. To test our prediction, additional experiments are required, e.g., we predict that tumor growth could be reduced by targeting the receptors of CAFs that are predicted to interact with the most abundant clones. This finding is not just of theoretical interest; it has important implications for treatment as it suggests that multiple clones may synergize in the TME and that more than one clone may need to be targeted.

One shortcoming of our study is that the number of cancer types, patients, and cells profiled is relatively small and that mutations are inferred from the transcriptome rather than the genome. Thus, it is likely that the observed clonal diversity is an underestimate since non-coding mutations are poorly represented. We also found a substantial number of unknown mutations from the COSMIC database in each cancer clone (Fig. 1C), indicating that there may be additional functional aspects that have been affected. The fact that we were only able to study two types of cancer with a limited number of patients and conditions means that one must be cautious about generalizing our findings. One reason for the narrow scope of our study is the scarcity of full-length scRNA-seq currently available in the public domain. The vast majority of scRNA-seq datasets were generated by 3′ end or 5′ end sequencing using the 10X Genomics platform. Unfortunately, SNV calling is much more challenging with this technology, and consequently it is hard to perform co-evolution analysis of genomic and transcriptomic with Canvolution. However, long-read sequencing technologies from PacBio [76] and Oxford Nanopore have recently been combined with single-cell DNA/RNA [77, 78], and we anticipate that it will be straightforward to apply Canvolution to this type of data.

Moreover, our data only offers a snapshot of the tumor, and this makes it difficult to ascertain saturation of clonal selection. Although there have been seminal studies employing CRISPR based lineage tracing with cancer cells [13, 14], it is much more challenging to apply these techniques to human cancers since they require genetic modifications. Naturally occurring mutations in the mitochondrial genome have been demonstrated to offer a powerful means for lineage tracing without genetic modifications, but they do not offer information about the mutations in the nuclear genome. A third issue is that we have only considered point mutations and short indels, ignoring large copy number variants which often are thought to have a bigger impact on the phenotype since they can change the gene dosage. However, several recent studies have focused only on small mutations and shown that they can have substantial impact on the TME [79], response to immunotherapy [80], and epithelial to mesenchymal transition [14]. These studies indicate that there are significant effects of point mutations and short indels, not only for the corresponding gene expression but also for the fitness of each clone. Fourth, an important finding from our study is that mutated proteins frequently have altered expression levels. This implies that one must take care in predicting the impact of changes in expression in a cancer cell as the function of the corresponding protein may differ from a healthy cell. In principle, mutations resulting in the loss or gain of function could also affect what gene signatures are enriched. However, given the difficulties of predicting the impact of a mutation on gene function, we have not taken this into consideration in our analyses.

## Conclusions

Despite these limitations, we believe that the overall trends are robust. Both metastasis and drug responsiveness were assessed by histology, and the drug resistant samples in LC were collected after a median of eight months. Thus, we believe that the classification of samples into different contexts is reliable, along with the scRNAseq profiling. In conclusion, our study of cancer evolution in human patients has revealed insights regarding the interactions of the mutational and transcriptional landscapes. These findings could be of clinical relevance as they suggest that one needs to target multiple clones that are cooperating in the TME to avoid the emergence of drug resistance.

## Methods

### Dataset

The dataset for lung cancer was obtained from Maynard, A. et al. [17], and chronic myeloid leukemia (CML) was obtained from Giustacchini, A. et al. [33]. We used normalized matrix, cell-type annotation, and metadata provided by the authors. We excluded “unknown” cells.

### Variant calling

We used Trinity Cancer Transcriptome Analysis Toolkit (CTAT) (v2.0.1) for the variant calling of cancer cells in scRNA-seq data [24]. It is based on the GATK Best Practices pipeline [81]. We matched “single-end” and “paired-end” for each dataset using default parameters without any boosting method (–boosting_method = none). We further filtered out non-COSMIC SNPs found in dbSNP and RNA-editing sites provided by CTAT with filtered.vcf files. During the process, 45/3754 LC cells and 36/1992 CML cells were excluded due to no confident SNV hits.

### Clonotyping

We merged the vcf files from the same patient by the “merge” function in bcftools (v1.11) with “—no-index, –missing-to-ref” options with default parameters [82]. After merging, we used DENDRO (v0.2.2) [7] to infer the clonotype for each cancer cell. The input mutation matrix derived from vcf files for DENDRO were obtained from our customized code based on the DENDRO package. The genetic divergence matrix was obtained by the negative-loglikelihood model using “DENDRO.dist” function with default parameters. Further kernel-based clustering was performed for grouping cells by clonotype using DENDRO.cluster with default parameters. During the mutational clustering profile, we modified the original code not to generate triple mutations from a single allele locus since ploidy was set to 2 (0: no mutation, 1: heterozygosity, 2: homozygosity). “optK” was defined for optimal “elbow point” using “DENDRO.icd” and “cutree” functions with default parameters for each patient to optimize the clustering. Finally, re-estimated mutation profile was obtained by adjusting “optK” from above using “DENDRO.recalculate” with default parameters.

### Subclonal evolution tree generation

We generated a subclonal evolution tree from the genotype information of each clonotype generated by DENDRO with RobustClone (no version) [5]. This approach orders clonotypes by a minimum-spanning tree algorithm. We obtained clonal tree by running “plot_MST” function using the mutational profile for each clone obtained from DENDRO with default parameters.

### Cancer cell clustering

We clustered cancer cells in each dataset using the Seurat package (v4.1.0) pipeline [26] with default parameters. We used the functions FindVariableFeatures, ScaleData, RunPCA, FindNeighbors (pc:1 ~ 30), and FindClusters (resolution: 1).

### Clonotype and cluster abundance analysis

For each sample, we used the proportion of each clonotype or cluster for abundance measurement. We measured the entropy of each clonotype or cluster of cancer cells by evaluating normalized Shannon entropy from Chazarra-Gil, R. et al. (no version) [83]. We calculated the fraction of clusters found in each clonotype, and we used these probabilities to obtain the Shannon entropy. To allow comparison across samples, we normalize by the theoretical maximum entropy for the given number of clusters in each sample. If a given clonotype has one cluster, we assign entropy as 0. We followed the same strategy for the clusters. Normalized mutual information between cancer clone and cluster was measured by “NMI” function in “aricode; v.1.0.2” package using “variant” parameter for “sqrt.”

### Signature gene set

We obtained fitness genes from Behan et al. including lung cancer and core fitness gene set [29] and cancer hallmark gene sets from CancerSEA [27]. We obtained the driver gene, oncogene, and tumor suppressor gene for lung cancer and CML from CancerMine [28]. All subtypes were merged for the corresponding tumor type. We obtained the cancer-testis gene from Wang et al. for lung cancer [30]. For metabolic pathways, we obtained human metabolic pathways from KEGG (hsa01100) [31].

### Mutation signature score

We measured the Jaccard index (intersection/union) between the mutation profile of a given clonotype and the signature gene set for signature scoring. We only considered the consensus CDS (CCDS) for protein-coding genes with “public availability” [84].

### Transcription signature score

We used the “AddModuleScore” function in the Seurat package (v4.1.0) with default parameter settings [26]. For the metabolic pathway in KEGG, we excluded a pathway if fewer than three genes were shared.

### Mutated gene expression signature score

Firstly, we defined mutation profiles from each feature from the “Signature gene set” section. Then, we average the gene expression level only for the mutated genes in a given signature gene set. For the metabolic pathway in KEGG, we excluded a pathway if fewer than three of the genes were expressed.

### CellChat

We inferred cell–cell interaction using the CellChat package (v1.6.1) [32] with only lung cancer data. Firstly, we inferred cell–cell interaction with different clusters of cancer cells (by gene expression) by default pipeline of CellChat except “min.cells = 0” for the “filterCommunication.” Next, we dissected each cluster into the different origins of clonotype (hereafter: cluster_clones). We measured the Jaccard index between each mutation profile of cluster_clones and all the genes included in the interactions (distinguished by target or source of cancer clusters) between certain cell types. During this process, we only considered CCDS genes [84]. For the interaction between tumor cells, we merged all samples.

### Mutated-LR score

The LR-signature score was evaluated for each clone using

$$\sum_{i}{n}_{i}*{jaccard\_index}_{i}/{n}_{clone}$$

*i*: clone_cluster (cluster within a given clone), *n*: number of the cell, ${n}_{clone}$: number of the cell in a given clone

### Evolutionary path analysis

This analysis was conducted for each sample. For the evolution path in each sample, we excluded clones with fewer than ten cells. From each evolution path, we evaluated Spearman correlation coefficients of mutation signature score, transcription signature score, mutated gene expression signature score, or cell–cell interaction score compared to the tree depth.

### Clonal selection analysis

This process was conducted in the same way same as the evolutionary path analysis, except we used relative clonal abundance instead of the tree depth for the correlation analysis.

### Simulation method

To simulate the data, we adopted from oncoNEM [85] (v.1.0). We first generate a set of genotypes by randomly assigning mutations. Next, a clonal tree is constructed from the synthetic genotypes. Then, simulated cells are created from the clonal tree with user-specified noise levels. In our simulations, we used the default settings for “oncoNEM” function (FPR: 0.2, FNR: 0.1, missing ratio: 0.2). To test our “evolutionary path analysis,” we generated a perfect signature correlated with the ground truth clonal tree (i.e., each level assigned the value 1, 2, 3, etc.) and a random signature (i.e., each level assigned a random number). We then calculated the Spearman correlation coefficient of the perfect signature and the random signature by our method (evolutionary path analysis) using the noisy clonal trees.

For “clonal selection analysis,” we used a similar strategy whereby we generated a signature which is perfectly correlated with the abundance of clones from the ground truth tree and a random signature. We also obtained noisy abundance estimates using the same simulation as above. Then, we obtained the Spearman correlation coefficient of the perfect and random signatures by our method (clonal selection analysis).

For the simulation scheme, we altered the number of cells, number of clones, and number of mutation sites with 100 iterations for each combination of parameters.

### oncoNEM function usage

1. simulateData (generates the goldstandard clonal tree and simulates a genotype matrix with a noise level)
  Parameters
    Cell: N.clones = 5, N.sites = 100, N.unobs =0, FPR = 0.2, FNR = 0.2, p.missing = 0.2
    Clone: N.cells = 20, N.sites = 100, N.unobs =0, FPR = 0.2, FNR = 0.2, p.missing = 0.2
    Mutation site: N.cells = 20, N.clones = 5, N.unobs =0, FPR = 0.2, FNR = 0.2, p.missing = 0.2
2. oncoNEM, search, clusterOncoNEM (generates a clonal tree from the simulated genotype).
  Parameters
    delta = 50, epsilon = 10 (default parameter setting)

We compared the Spearman correlation coefficient of the perfect and random signature by a Wilcoxon rank sum test (two-sided).

### Statistical analysis

We performed the Wilcoxon-rank sum test or the Kruskal–Wallis test between different groups of interest in each dataset with given features by either “evolutionary path analysis” or “clonal selection analysis.” We used the Benjamini–Hochberg procedure for the multiple-hypothesis correction.

### Differentially expressed gene analysis

We used the FindMarkers function implemented in the Seurat package (v4.1.0) with "logfc.threshold = 0.25” and “min.pct = 0.25″ and default values for the remaining parameters. We used p_val_adj < 0.01 for a significant gene.

### Significant feature overlapping analysis

To measure the overlap between significant features from mutation evolution analysis and transcription evolution analysis as quantified by the evolutionary path analysis and the clonal selection analysis, the observed overlap probability and expected overlap probability were calculated as follows:

- observed overlap probability: overlapping significant features/total significant features
- expected probability: (significant features from set1/total significant features) * (significant features from set2/total significant features)

Each feature with different enrichment of group (e.g., metastasis or primary) was counted separately. If there is no significant result from one of the comparison sets, the value is assigned 0. For drug response in lung cancer data, we collapsed all non-PD groups.

To measure the overlap between significant features for metastasis (LC), disease progression (LC), or poor response (CML) from transcription evolution analysis and mutated-gene expression analysis, observed overlap probability and expected overlap probability were calculated as follows:

- observed overlap probability: overlapping significant features/significant features in transcription evolution analysis
- expected probability: overlapping significant features from any of the group/significant features from any of the groups in transcription evolution analysis

### Literature search

We searched each feature for the corresponding condition (e.g., metastasis, drug resistance) if any cancer type shows a relevant relationship in the literature using PubMed. If we were unable to find relevant work, we counted it as a false positive (FP). We also counted it as FP if the enrichment of the pathway or metabolism is the opposite direction of the literature consensus. We also counted “true” if the feature is relevant by tautology (e.g., metastasis signature for metastatic cancer).

### TCGA data analysis

We collected RSEM normalized expression data, clinical data, and mutation_packager_oncotated_call data of lung adenocarcinoma (LUAD) and lung squamous cell carcinoma (LUSC) from The Cancer Genome Atlas (TCGA (https://gdac.broadinstitute.org/). We only collected patients who had chemotherapy clinical data. For inferring mutated ligand-receptor analysis, we merged all the ligands or receptors of cancer cells from CellChat results for each target cell. Then, we measured the Jaccard index between those results and mutations in each sample. Patient samples were then split into a high and a low group based on the median of the Jaccard index. We used progression-free interval (PFI) survival information from elsewhere [73]. We used Kaplan–Meier curves for survival analysis with a given feature divided by the “high” or “low” group by its mean value.

### Supplementary Information

**Additional file 1.** Supplementary Information. Supplementary Information for Figure S1 ~ S9.

**Additional file 2: Table S1.** Sample information and statistics for mutation.

**Additional file 3: Table S2.** Mutation frequency results.

**Additional file 4: Table S3.** Raw statistical results of Canvolution in LC and CML.

**Additional file 5: Table S4.** Accession of literature for validating Canvolution (cancer signature).

**Additional file 6: Table S5.** Accession of literature for validating Canvolution (Metabolism).

**Additional file 7: Table S6.** Result of survival analysis.

**Additional file 8.** Review history.

## Data Availability

Code availability: Canvolution was developed in R and it is available at (https://github.com/jaewon-cho/canvolution/tree/master) [86]. This GitHub is licensed under the MIT license. Raw scripts for the paper can be found from 10.5281/zenodo.10642609 [87]. This Zenodo is licensed under a Creative Commons Attribution 4.0 International. Data availability: The lung cancer dataset was obtained from an NCBI BioProject #PRJNA591860 [17]. Chronic myeloid leukemia (CML) was obtained from SRP067759 and GSE76312 [33]. TCGA data was obtained from https://gdac.broadinstitute.org/.
