# Supplementary material for: Joint analysis of mutational and transcriptional landscapes in human cancer reveals key perturbations during cancer evolution
Source: Genome Biol. 2024 Mar 8;25:65. doi: 10.1186/s13059-024-03201-1 (PMC10921788; doi:10.1186/s13059-024-03201-1)
Supplement: Supplementary file 1 — Additional file 1. Supplementary Information. Supplementary Information for Figure S1 ~ S9. [file 13059_2024_3201_MOESM1_ESM.docx]

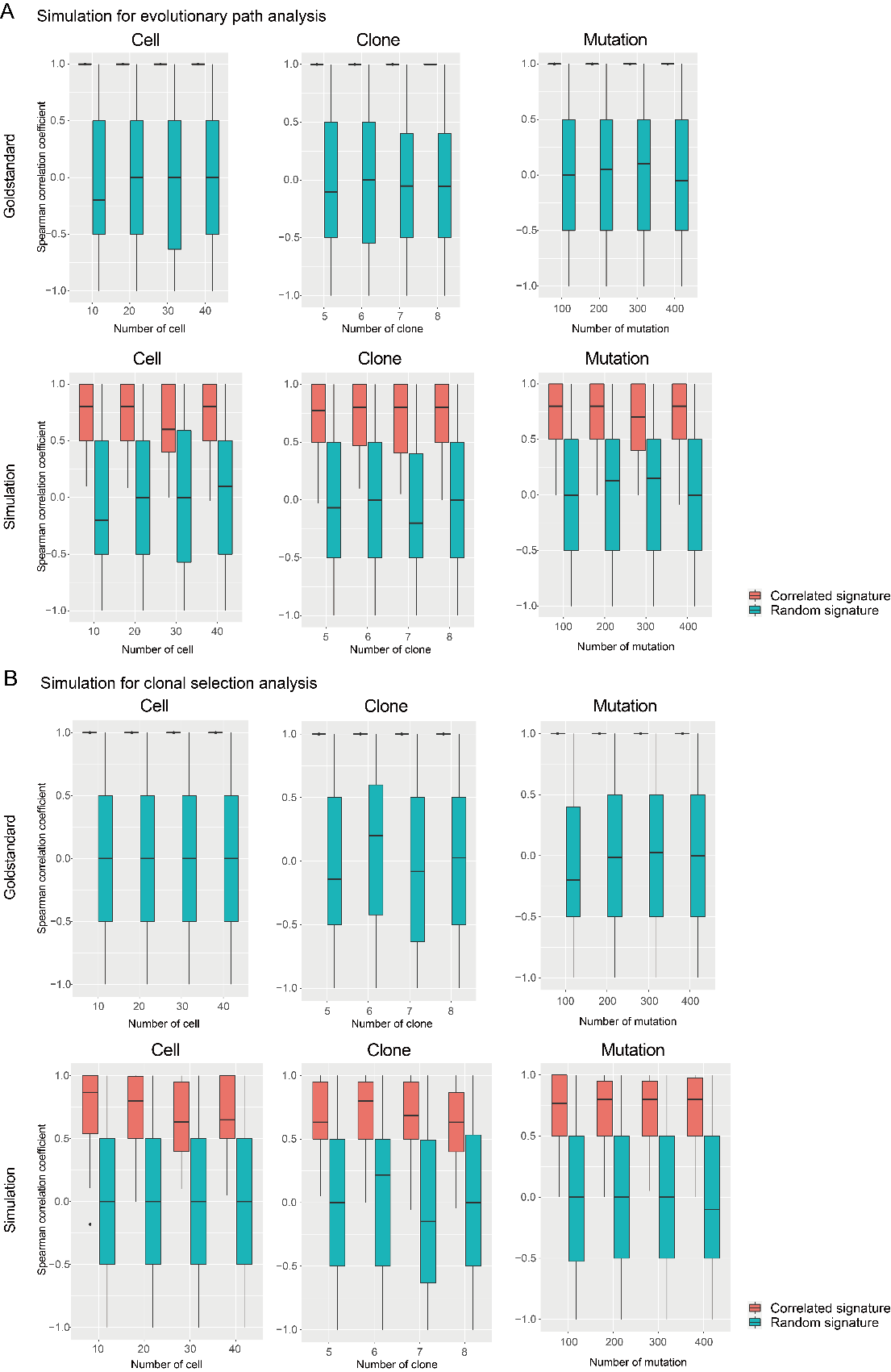


Figure S1. Evolutionary path analysis (A) and clonal selection analysis (B) for synthetic datasets. The ground truth result (top) indicates the correlation of the perfect and the random signatures. The simulation result (bottom) indicates the correlation of the perfect and the random signatures. Each panel indicates the alteration of cell count (left), number of clones (middle), and number of mutations (right) during the simulations. Each box corresponds to n=100 simulations. The perfect and the random signature scores were compared and P values were obtained by Wilcoxon-rank sum test (two-sided). All the p values were < 1E-26.


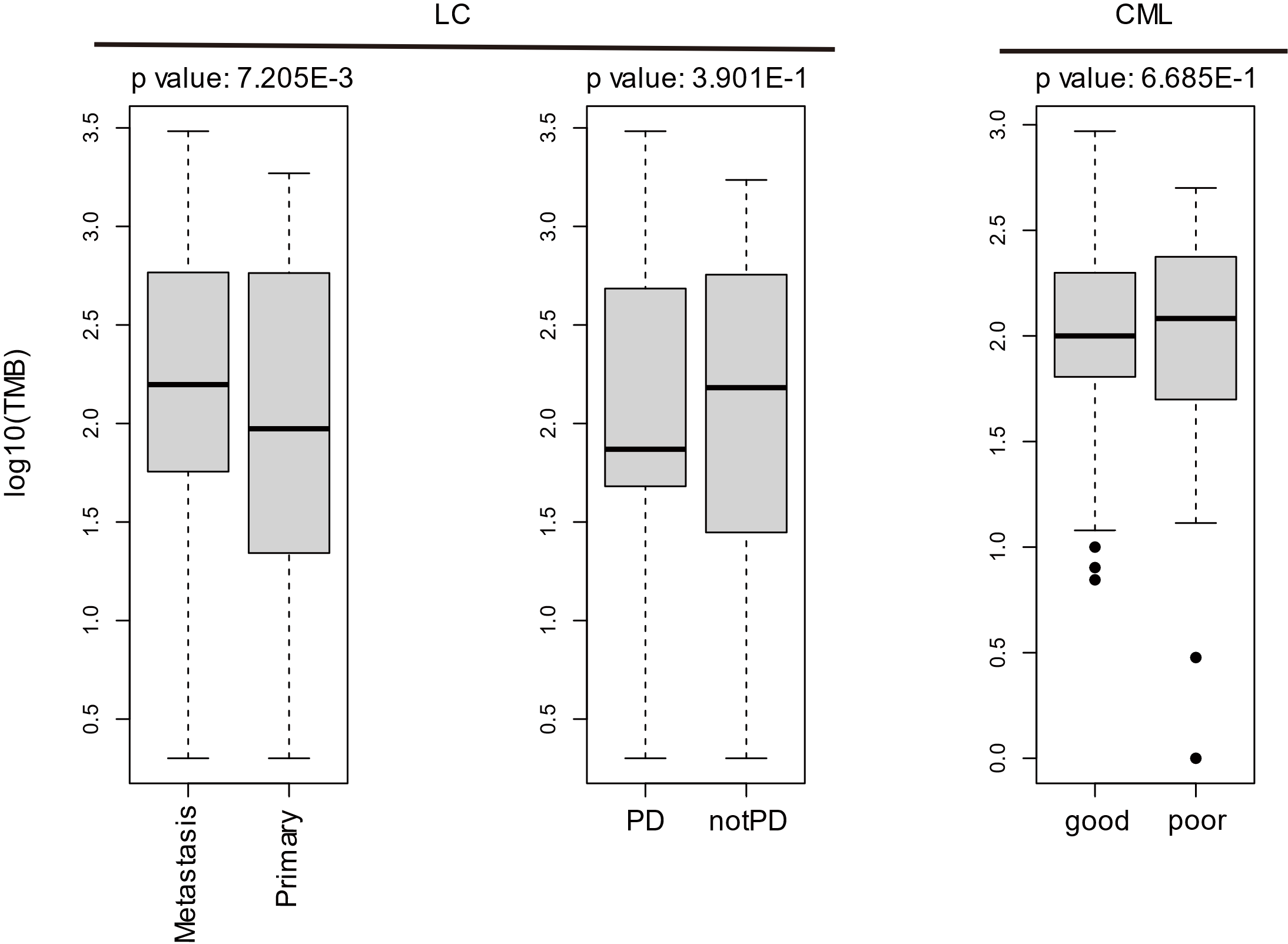


Figure S2. Boxplots showing the TMB between two groups. P value was obtained by Wilcoxon-rank sum test (two sided).


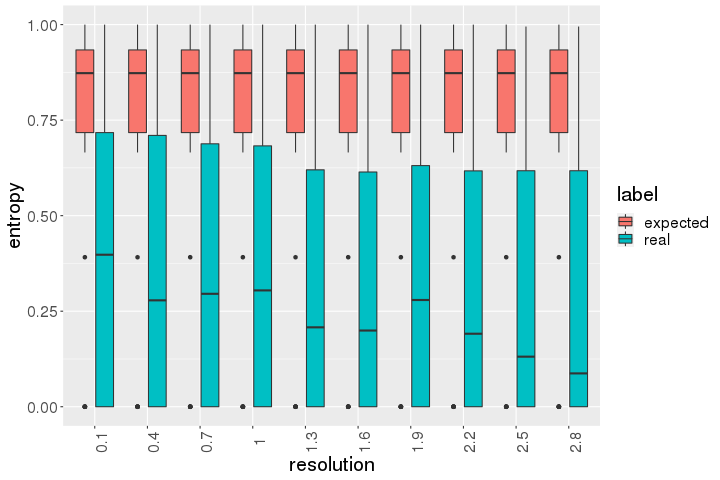


- LC


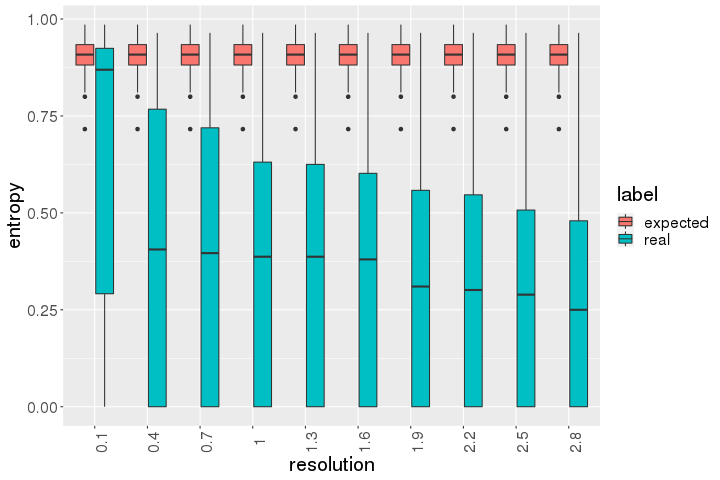


- CML

Figure S3. Cluster entropy across the different resolutions of clustering


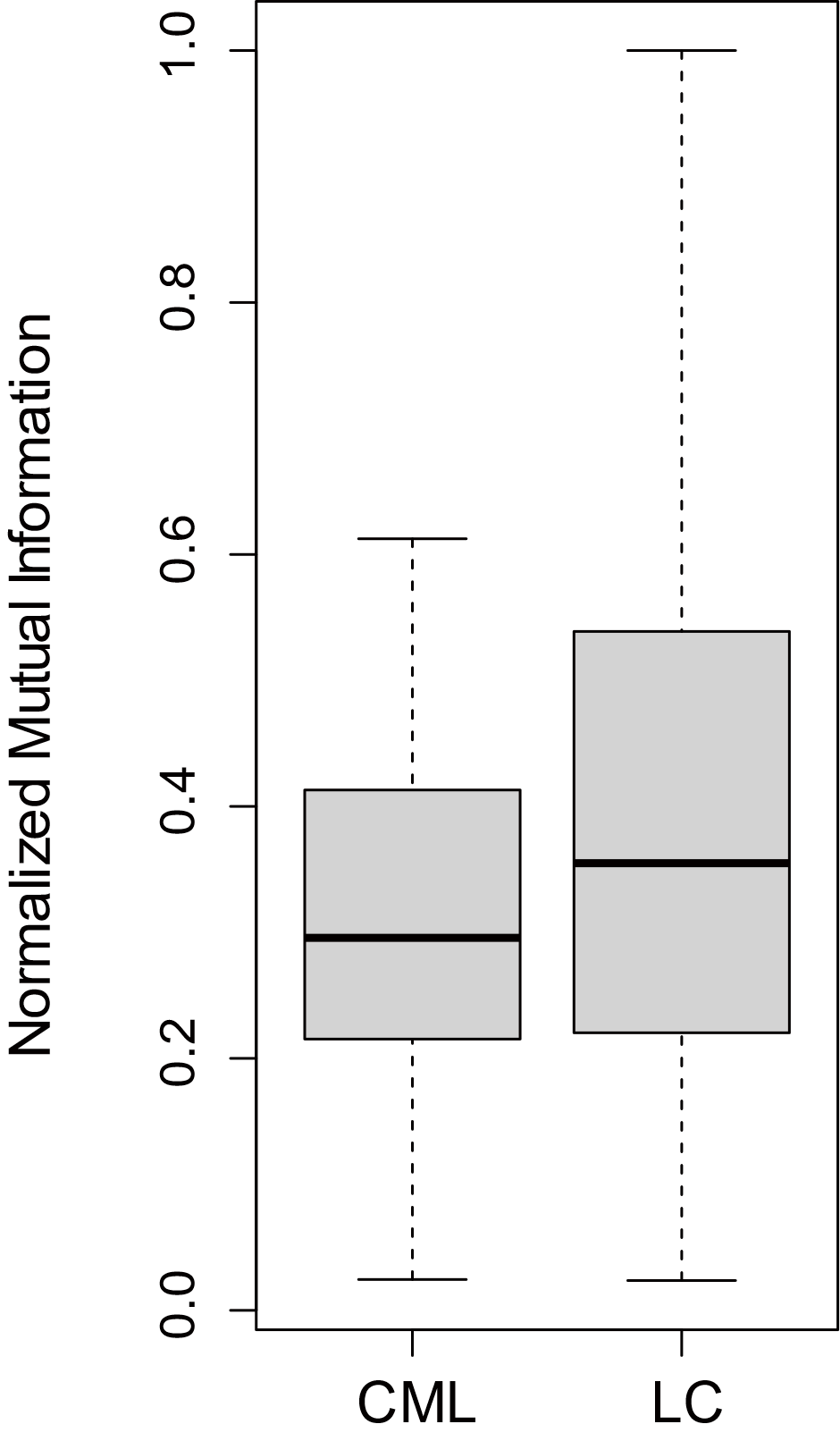


Figure S4. Boxplots showing the normalized mutual information between cancer clone and cluster in each sample for CML and LC.


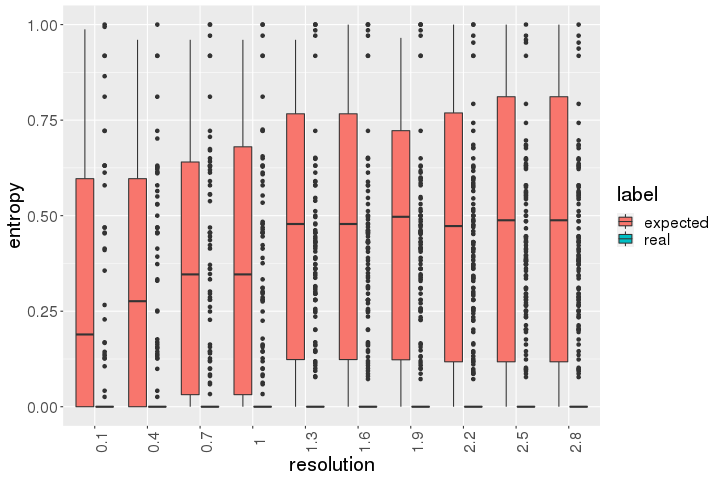


- LC


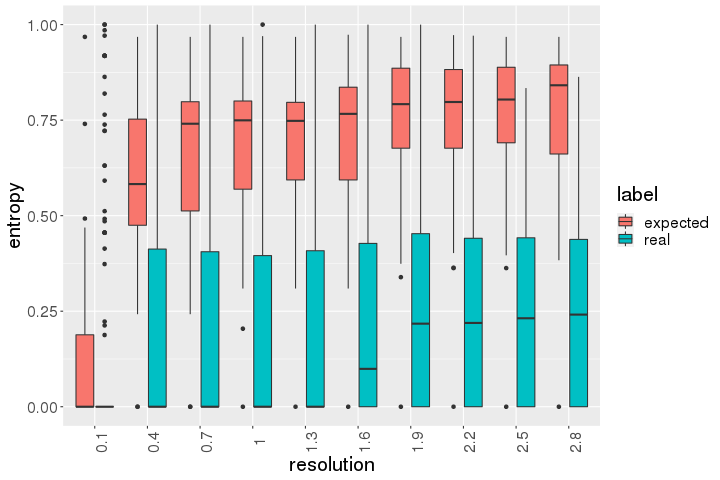


- CML

Figure S5. Clonal entropy across the different resolutions of clustering


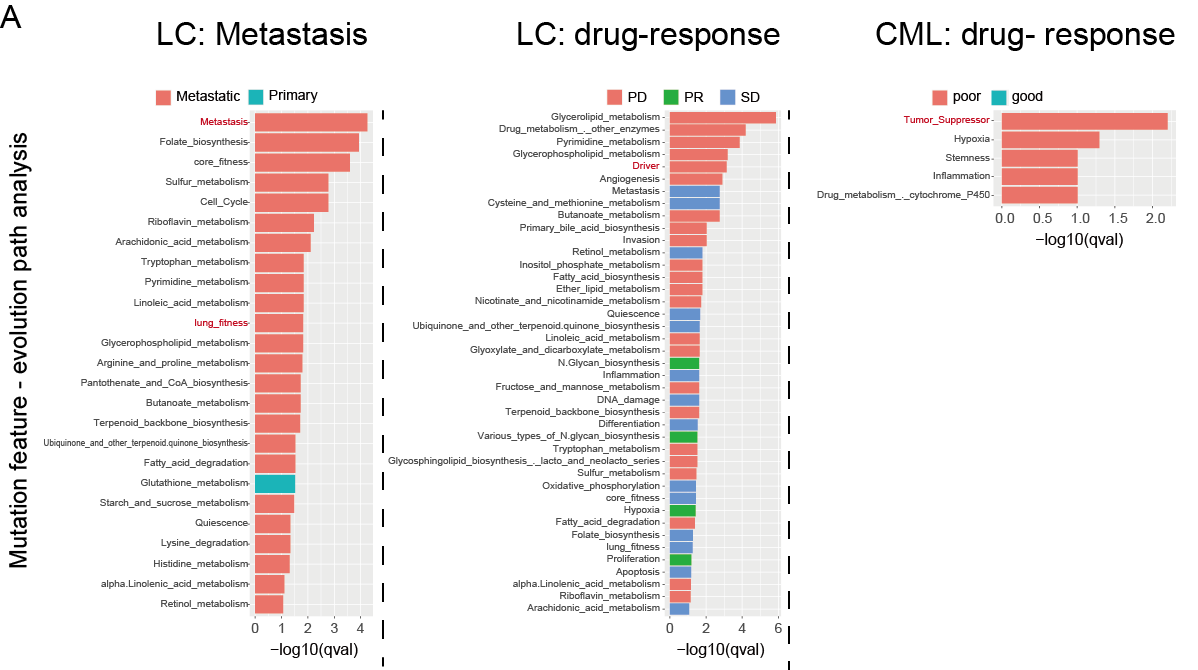


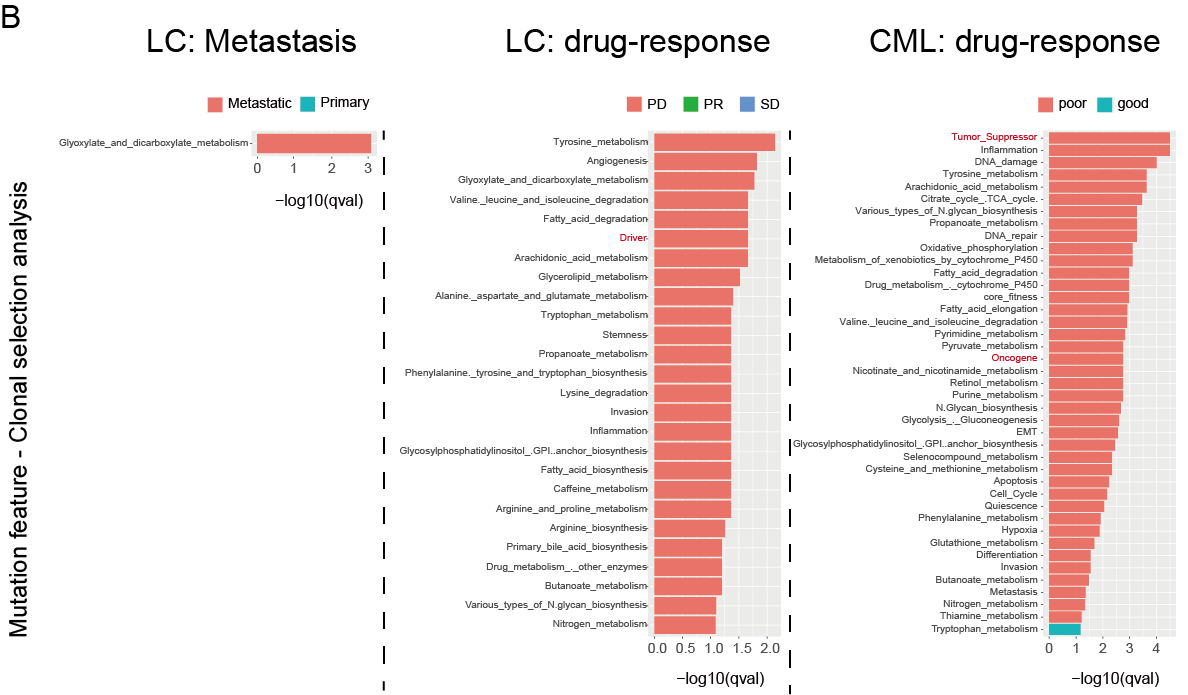


Figure S6. Evolution and selection analysis of mutation features (LC, CML)

(A) The horizontal bar plots of evolution path analysis with mutation features. (B) The horizontal bar plots of clonal selection analysis with mutation features. “metastasis” versus “primary” (left panel) and “progression disease (PD),” “partial response (PR),” and “stable disease (SD)” in lung cancer (middle panel). “good response” and “poor response” in CML sampled at diagnosis (right panel). Only the q-value <= 0.1 was shown. The bar's color indicates the group's highest mean value during group comparison.


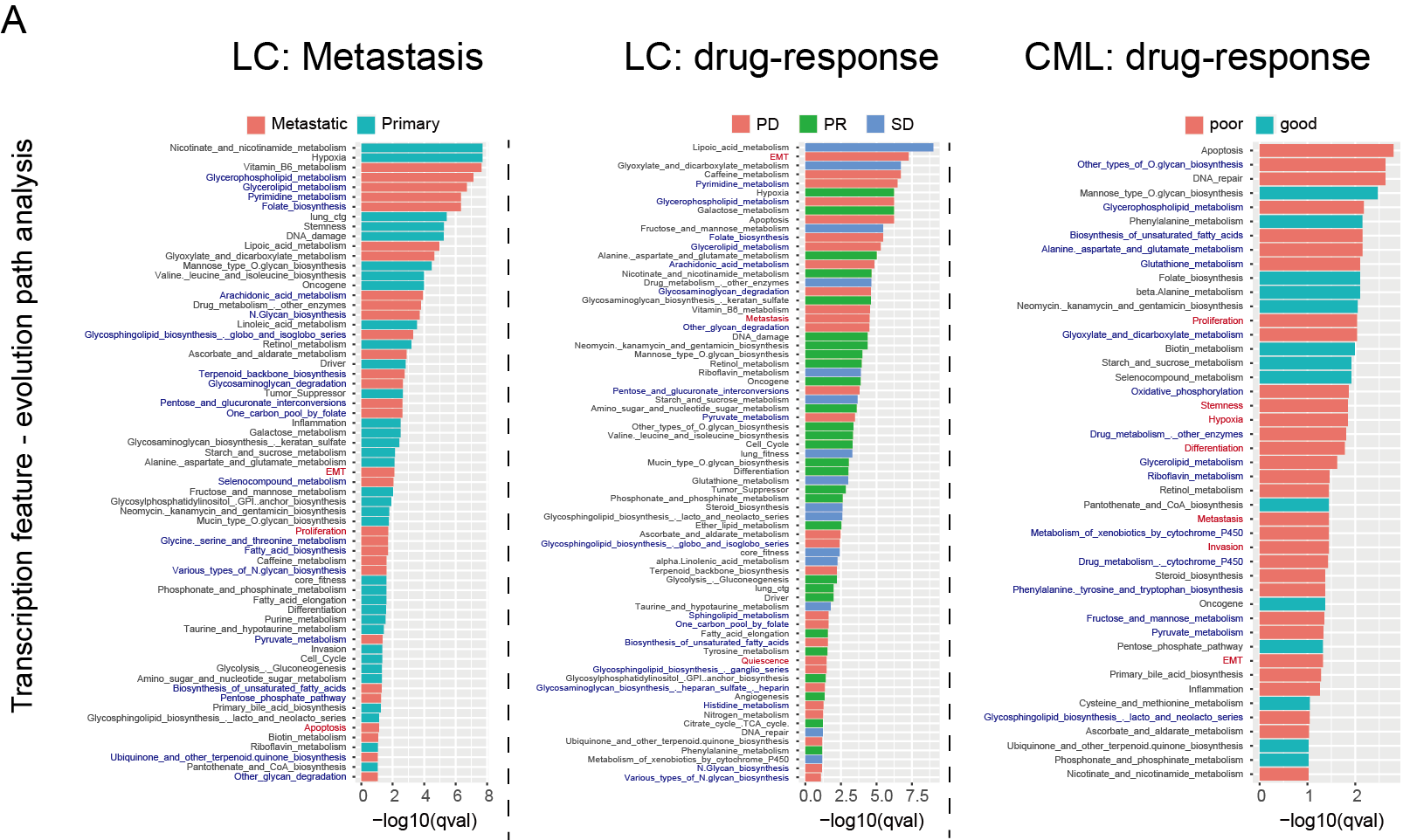


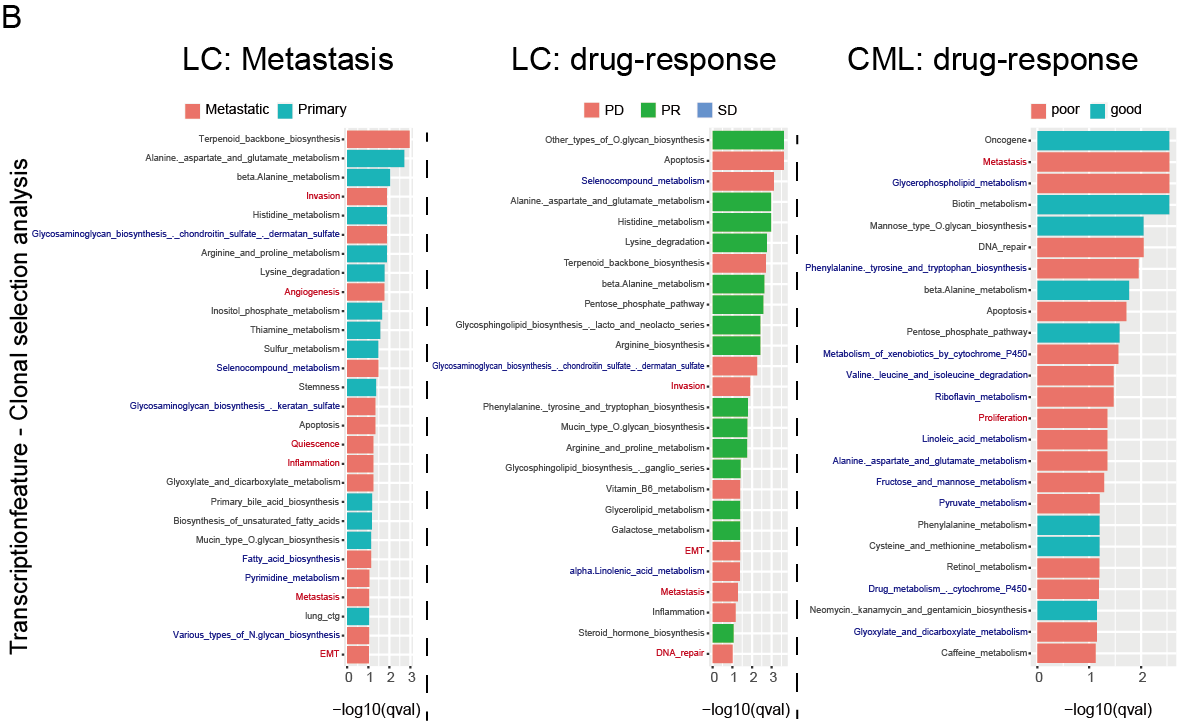


Figure S7. Evolution and selection analysis of transcription features (LC, CML)

(A) The horizontal bar plots of evolution path analysis with transcription features. (B) The horizontal bar plots of clonal selection analysis with transcription features. “metastasis” versus “primary” (left panel) and “progression disease (PD),” “partial response (PR),” and “stable disease (SD)” in lung cancer (middle panel). “good response” and “poor response” in CML sampled at diagnosis (right panel). Only the q-value <= 0.1 was shown. The bar's color indicates the group's highest mean value during group comparison.


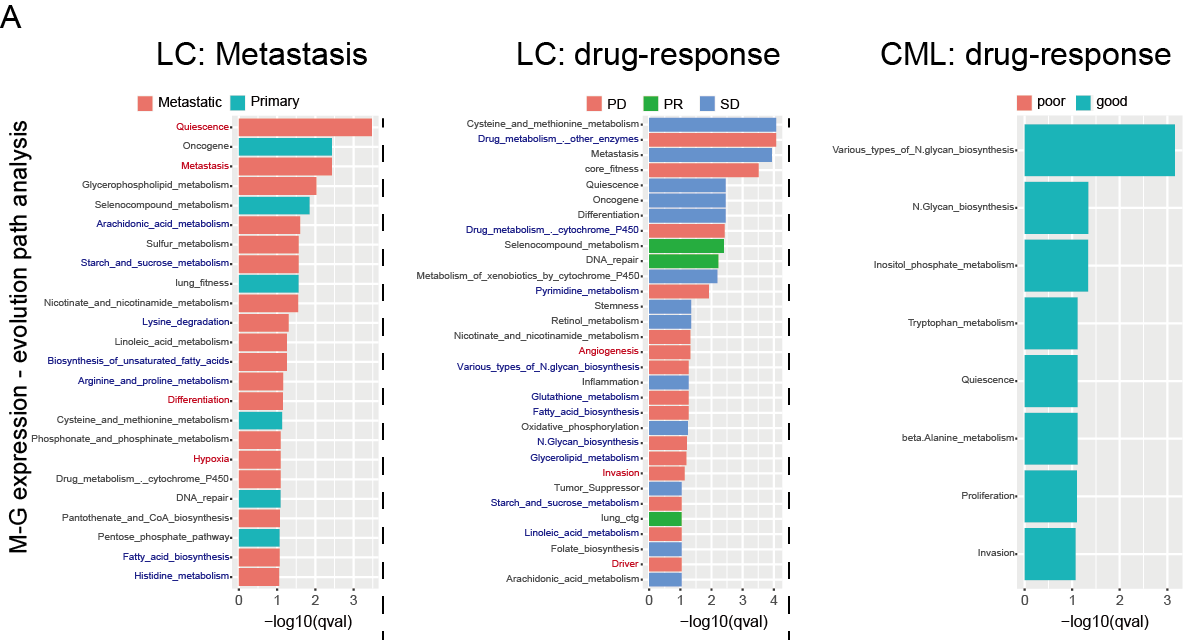


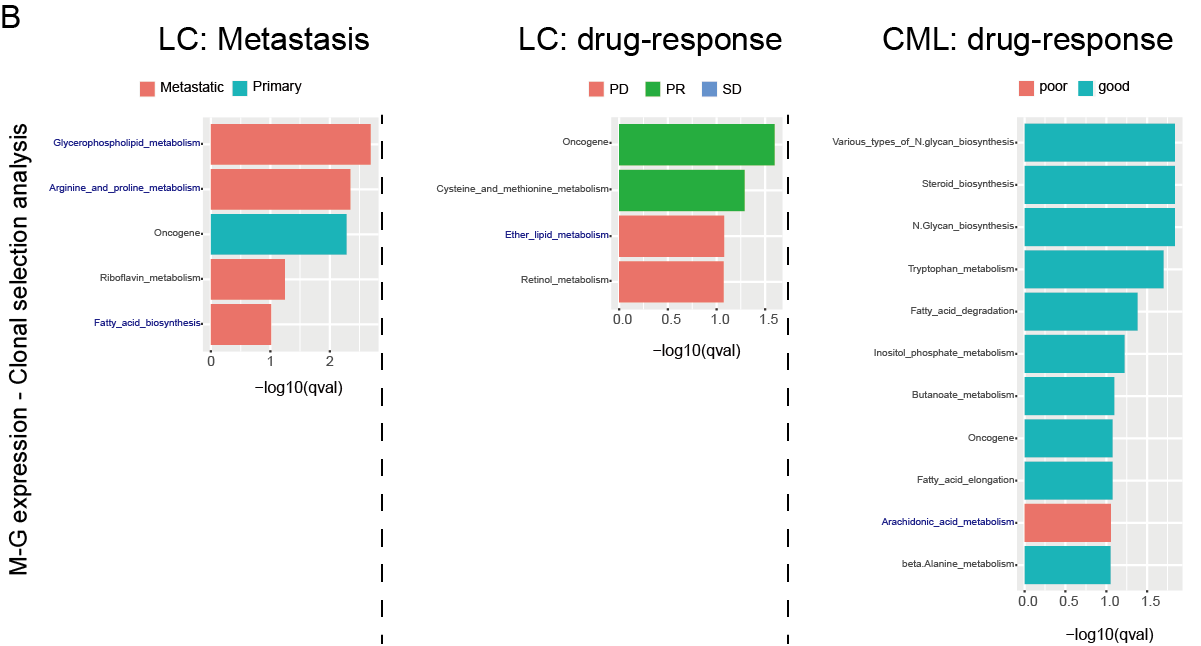


Figure S8. Evolution and selection analysis of mutated gene expression (LC, CML)

(A) The horizontal bar plots of evolution path analysis with mutated gene expression. (B) The horizontal bar plots of clonal selection analysis with mutated gene expression. “metastasis” versus “primary” (left panel) and “progression disease (PD),” “partial response (PR),” and “stable disease (SD)” in lung cancer (middle panel). “good response” and “poor response” in CML sampled at diagnosis (right panel). Only the q-value <= 0.1 was shown. The bar's color indicates the group's highest mean value during group comparison.


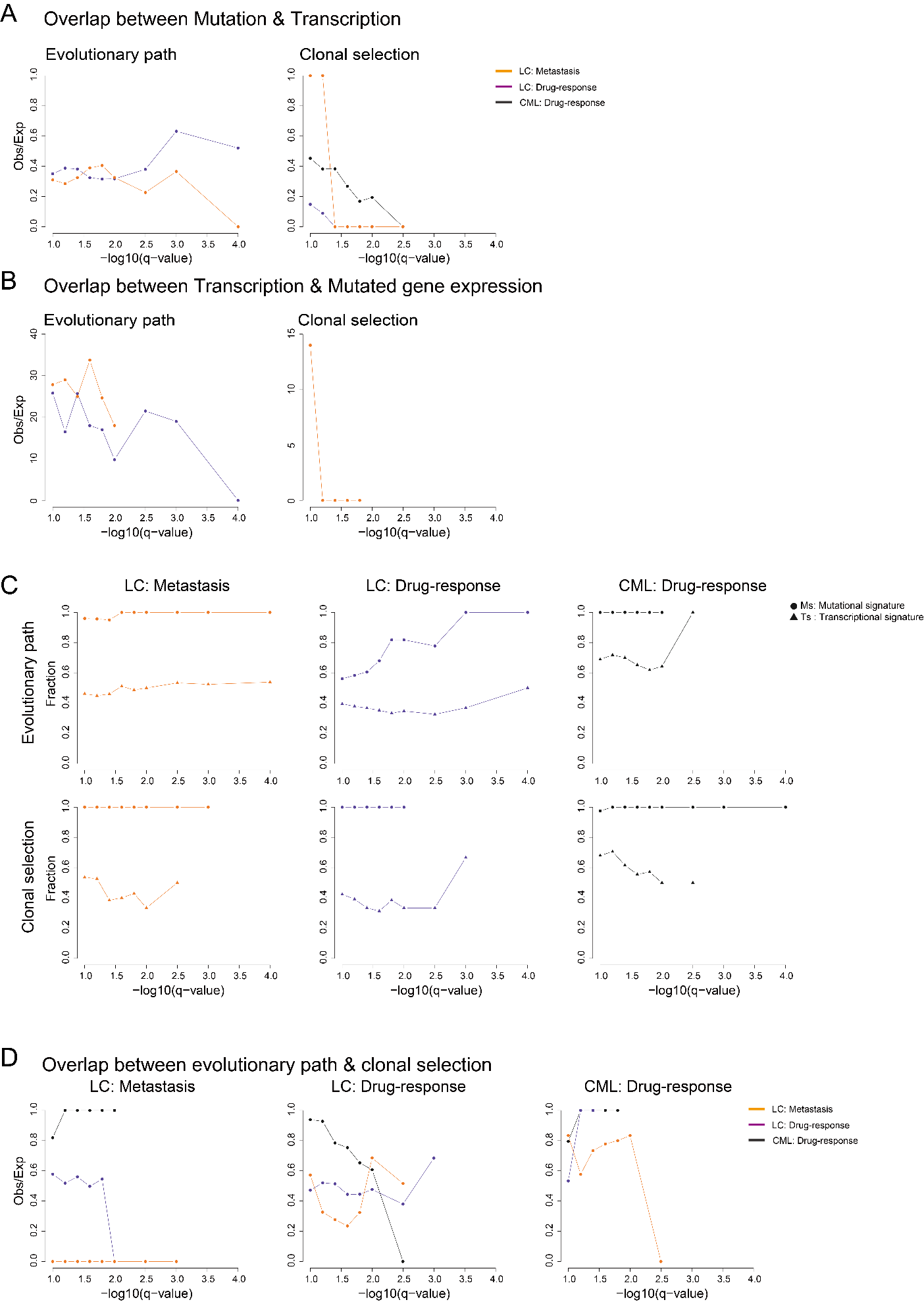


Figure S9. Robustness of the analysis (A) The overlap of features between mutational analysis and transcriptional analysis in the evolutionary path and clonal selection analyses with different q-value thresholds (related to Figure 5D). (B) The overlap of features between transcriptional analysis from the “metastasis” or “PD” group and mutated gene expression analysis in the evolutionary path and clonal selection analyses with different q-value thresholds (related to Figure 5E). (C)The fraction of features from “metastasis,” “PD,” or “poor response” in mutational signature and transcriptional signature with different q-value thresholds (related to Figure 5F). (D) The overlap of features in the same enrichment group (mutational signature, transcriptional signature, and mutated gene expression) between the evolution path analysis and clonal selection analysis with different q-value thresholds (related to Figure 5G).
