# Supplementary material for: Joint analysis of mutational and transcriptional landscapes in human cancer reveals key perturbations during cancer evolution
Source: Genome Biol. 2024 Mar 8;25:65. doi: 10.1186/s13059-024-03201-1 (PMC10921788; doi:10.1186/s13059-024-03201-1)
Supplement: Supplementary file 8 — Additional file 8. Review history. [file 13059_2024_3201_MOESM8_ESM.docx]

Review History

**First round of review**

**Reviewer 1**

**Were you able to assess all statistics in the manuscript, including the appropriateness of statistical tests used?**

Yes, and I have assessed the statistics in my report.

**Comments to author:**

In this study, Cho et al. developed a bioinformatics pipeline named Canvolution, with the primary goal of analyzing cancer evolution and clonal selection. The mutational and transcriptional profiles obtained from individual cells are integrated. The researchers applied this pipeline to two publicly available single-cell RNA-seq datasets, originating from patients afflicted with either lung cancer or chronic myeloid leukemia. While this study doesn't introduce groundbreaking methodological innovations, it does offer novel biological insights, particularly through its pioneering characterization of human tumor patients. This characterization is achieved by simultaneously examining genetic and transcriptional profiles within the same cells. The study investigates how genetic and transcriptional disruptions in tumor cells might impact their fitness within the tumor microenvironment. Additionally, the research identifies specific mutations that correlate with the transcriptional statuses of related genes and mutations that affect ligand-receptor interactions. These findings underscore the potential to provide a innovative perspective on tumor evolution and enhance our understanding of tumorigenesis. However, there are certain concerns that should be addressed:

1. The authors should include an evaluation of Canvolution's performance using simulated datasets.

2. It is essential to determine whether there are any bioinformatics tools with identical or similar functionalities to Canvolution, regardless of their application to single-cell or bulk-cell datasets. If yes, a benchmark between Canvolution and these existing tools should be conducted. If no, the authors should clarify this in the Introduction or Discussion sections.

3. It is crucial to analyze whether the conclusions in this study significantly depend on the statistical cutoffs. A comprehensive analysis, at least for parts of the results, is necessary to demonstrate the robustness of these biological findings.

4. Another limitation of this study and Canvolution that should be discussed is the relative scarcity of full-length single-cell RNA-seq datasets compared to the abundance of 3' end or 5' end single-cell RNA-seq datasets (such as those from 10X Genomics).

**Reviewer 2**

**Were you able to assess all statistics in the manuscript, including the appropriateness of statistical tests used?**

Yes, and I have assessed the statistics in my report.

**Comments to author:**

In this study, Cho and Hemberg introduce a new tool, Canvoltion, that is able to integrate cancer evolution and clonal selection analyses alongside transcriptional profiling using full-length single-cell RNA-seq data. The workflow joins together multiple pieces of software for mutation calling, clonal inference and tree reconstruction, gene signature enrichment and ligand-receptor interaction inference. The power of the method is highlighted on two publicly available datasets from lung cancer and chronic myeloid leukemia with available treatment response annotation. The authors generally find high clonal and transcriptional diversity linked to drug resistance, which leads them to posit that clonal sweeps are unlikely to be common during the evolution of these cancers. This original approach at inferring joint mutational and transcriptional evolution in single cell data illustrates how information from single cells can be maximised to understand cancer evolution, and can have many valuable applications in the field. The authors make the code freely available and a good tutorial is provided for using the tool on Github. The statistics employed in the study are appropriate, although not all of the metrics illustrated in the figures are entirely intuitive and could use more explanations in the figure captions. I have the following comments/suggestions that I believe would improve the clarity and robustness of the study:

1. A more detailed description of the CML and LC datasets employed in the study would be helpful - e.g. how many good and poor responders are in each category? The authors highlight lack of significant differences between good and poor responders in several cases, but are they powered to identify such significant differences (assuming transcriptional signatures and clonal profiles are summarised per patient rather than per cell).

2. A higher clonal diversity was observed in donors with poor response to treatment in CML samples, but not LC samples. How can this discrepancy be explained?

3. "There were no significant differences in transcriptional diversity between the different conditions, arguing against the hypothesis that higher fitness of a tumor is associated with a more diverse mix of transcriptional states." - This is a bit of a strong statement, considering that pre-defined and fairly generic transcriptional signatures are employed. What if the signatures that are truly variable have been missed in this analysis? If the authors were to conduct an unbiased transcriptional analysis (e.g. using differential expression methods or similar), would there be no transcriptional signals arising as distinct between conditions?

4. Figures 5D-E: it is not clear to me what these two figures are showing, in particular what is represented on the x axis. Please detail abbreviations in the figure captions, and if possible make the labels more explicit (please do this for all figure panels where labels are cryptic). Can the authors explain this analysis in a bit more detail as well?

5. How generalisable are the findings in these two cancers to other types of cancer? While some similarities were pointed out, there were also some differences between CML and LC, so it is not entirely clear to what extent these findings are tissue and treatment specific.

6. Linked to the point above, I am not sure the following statement in the abstract can generalised to other cancers "This suggests that selection based solely on growth rate is unlikely to be a major driving force during cancer evolution.", and I am also not convinced the authors have provided sufficient evidence for clonal growth not being a major driver, considering the limitations of single cell sequencing experiments analysed (e.g. only surviving cells are sampled during/after treatment, but lack of lineage tracing means a change in phenotype from one time point to the other cannot be captured; lack of spatial resolution etc.).

7. The findings hinge on the assumption that clonal reconstruction from single cell RNA-seq data is accurate enough. The authors acknowledge the limitations in calling mutations from single cell RNA-seq data, but it would be helpful to have a sense of how the metrics would look like if mutations were called entirely randomly. Would the reconstructed trees be significantly different? How much of a dependency is there between the clonal reconstruction from inferred mutation data and the transcriptional signatures analysed?

8. The general aesthetics of the figures need improvement - spell out fully the axis labels without abbreviations, minimize white space etc.

**Authors Response**

**Point-by-point responses to the reviewers’ comments:**

Reviewer #1:

In this study, Cho et al. developed a bioinformatics pipeline named Canvolution, with the primary goal of analyzing cancer evolution and clonal selection. The mutational and transcriptional profiles obtained from individual cells are integrated. The researchers applied this pipeline to two publicly available single-cell RNA-seq datasets, originating from patients afflicted with either lung cancer or chronic myeloid leukemia. While this study doesn't introduce groundbreaking methodological innovations, it does offer novel biological insights, particularly through its pioneering characterization of human tumor patients. This characterization is achieved by simultaneously examining genetic and transcriptional profiles within the same cells. The study investigates how genetic and transcriptional disruptions in tumor cells might impact their fitness within the tumor microenvironment. Additionally, the research identifies specific mutations that correlate with the transcriptional statuses of related genes and mutations that affect ligand-receptor interactions. These findings underscore the potential to provide a innovative perspective on tumor evolution and enhance our understanding of tumorigenesis. However, there are certain concerns that should be addressed:

1. The authors should include an evaluation of Canvolution's performance using simulated datasets.

*Response: Thank you for the comment. We have evaluated the performance of the “evolutionary path analysis” and “clonal selection analysis” with simulated datasets since they form the basis of all downstream analyses. We adopted the simulation model from “RobustClone” (Chen et al, Bioinformatics, 2020) which we have used for building clonal trees. This synthetic data was generated by a simulation method from oncoNEM (Edith et al, Genome Biology, 2016). Briefly, we first generate a set of genotypes by randomly assigning mutations. Next, a clonal tree is constructed from the synthetic genotypes. Then, simulated cells are created from the clonal tree with user-specified noise levels. In our simulations we used the default settings for oncoNEM (FPR: 0.2, FNR: 0.1, missing ratio: 0.2). To test our “evolutionary path analysis”, we generated a perfect signature correlated with the ground truth clonal tree (i.e. each level assigned the value 1, 2, 3, …) and a random signature (i.e. each level assigned a random number). We then calculated the Spearman correlation coefficient of the perfect signature and the random signature by our method (evolutionary path analysis) using the noisy clonal trees.*

*For “clonal selection analysis”, we used a similar strategy whereby we generated a signature which is perfectly correlated with the abundance of clones from the ground truth tree, and a random signature. We also obtained noisy abundance estimates using the same simulation as above. Then, we obtained the Spearman correlation coefficient of the perfect and random signatures by our clonal selection analysis method.*

*For the simulations, we altered the number of cells, number of clones, and number of mutation sites, and for each combination of parameters we generated 100 realizations. Reassuringly, the perfect signatures were highly correlated compared to the random signature (all the p-value < 1E-26; Wilcoxon rank sum test, two-sided) from the simulated datasets.*

*Function 1) simulateData (generates the ground truth clonal tree and simulates a genotype matrix with a user specified noise level)*

*Parameters Cell: N.clones = 5, N.sites = 100, N.unobs =0, FPR = 0.2, FNR = 0.2, p.missing = 0.2*

*Clone: N.cells = 20, N.sites = 100, N.unobs =0, FPR = 0.2, FNR = 0.2, p.missing = 0.2*

*Mutation site: N.cells = 20, N.clones = 5, N.unobs =0, FPR = 0.2, FNR = 0.2, p.missing = 0.2*

*2) oncoNEM, search, clusterOncoNEM (generates a clonal tree from the simulated genotype)*

*Parameters*

*delta = 50, epsilon = 10 (default parameter setting)*

*Figure S1. Evolutionary path analysis (A) and clonal selection analysis (B) for synthetic datasets. The ground truth result (top) indicates the correlation of the perfect and the random signatures. The simulation result (bottom) indicates the correlation of the perfect and the random signatures. Each panel indicates the alteration of cell count (left), number of clones (middle), and number of mutations (right) during the simulations. Each box corresponds to n=100 simulations. The perfect and the random signature scores were compared and P values were obtained by Wilcoxon-rank sum test (two-sided). All the p values were < 1E-26.*

*We have included these results in the Results section on page 4, with further details in the Methods section and Supplementary Figure 1.*

2. It is essential to determine whether there are any bioinformatics tools with identical or similar functionalities to Canvolution, regardless of their application to single-cell or bulk-cell datasets. If yes, a benchmark between Canvolution and these existing tools should be conducted. If no, the authors should clarify this in the Introduction or Discussion sections.

*Response: Thank you for the comment. To the best of our knowledge, Canvolution is the first pipeline for joint analysis of genomic and transcriptomic in the single-cell resolution using patient data. Thus, we are not aware of any directly comparable tools. However, below we discuss some methods that partially overlap with Canvolution.*

*We could not find any related research using bulk data since all of them were limited to the evolutionary tree construction or detecting when the gene is mutated. The most similar method we could find was “Evolutionary signatures of human cancers revealed via genomic analysis of over 35,000 patients” by Fontana et al (Nat Comms, 2023, https://doi.org/10.1038/s41467-023-41670-3). This study infers the clonal tree with scDNAseq data and identifies enriched SNV signature associated with outcome from survival data using a regularized Cox regression model (evoSigs function). The method requires survival information for each patient and it is much more limited than Canvolution with regards to what types of signatures it can identify.*

*There are also two papers from the Weissman group at MIT analyzing cancer evolution with lineage-tracing mouse models which is based on CRISPR technology (Quinn et al, Science, 2021, https://doi.org/10.1126/science.abc1944 and Yang et al, Cell, 2022, https://doi.org/10.1016/j.cell.2022.04.015). They used lineage tracing technology to obtain a “phylotime” to indicate how long a cell had been evolving. Clearly, this approach is not feasible with data from human patients since they cannot be engineered to support lineage tracing. In addition, they analyzed the associated transcriptional signatures using TradeSeq (Koen et al, Nat comm, 2020), which is based on a generalized additive model. Hence, we compared our “evolutionary path analysis” by running TradeSeq with the phylogenetic trees from our data for transcriptional signature analysis. However, only 5.73 % (11/192) paths from LC and 6.64 % (15/226) paths from CML were fitted with TradeSeq. We suspect that the main reason why TradeSeq failed was due to the small number of cells in our samples. Hence, it was not feasible to compare with TradeSeq.*

*Taken together, there are no other methods that can provide a meaningful comparison to Canvolution. We have provided an abbreviated version of the arguments above in our Discussion.*

*TradeSeq method*

*We followed the script provided on GitHub (https://github.com/mattjones315/KPTracerrelease; reproducibility/Figure5_S5/scripts/ run_tradeseq.r). Gene filtering by VISION:::filterGenesThreshold (10 % cells should express) was conducted for each dataset. The depth of the clone was used for pseudotime (phylotime for cancer evolution). “fitGAM” in “Tradeseq” package was conducted to obtain the gene associated with the phylotime.*

3. It is crucial to analyze whether the conclusions in this study significantly depend on the statistical cutoffs. A comprehensive analysis, at least for parts of the results, is necessary to demonstrate the robustness of these biological findings.

*Response: Thank you for the comment, we agree that robustness to statistical cut-offs is important. To address this issue we repeated several of our analyses: 1) overlap between mutational signature and transcriptional signature in Fig. 5D*

*2) overlap between transcriptional signature and mutated gene expression in Fig. 5E*

*3) fraction of mutational signature or transcriptional signature in the advanced phenotype (LC metastasis: Metastatis group, LC drug response: PD, CML drug response: poor) in Fig. 5F*

*4) Overlap between evolutionary path and clonal selection analysis in Fig. 5G, with q value thresholds (-log10[q-value]: 1, 1.2, 1.4, 1.6, 1.8, 2, 2.5, 3, 4).*

*Reassuringly, the results were consistent with what was reported in the main figure of the manuscript. The results are shown in the new Fig S9 and in summary we find:*

*1) The overlap between mutational signature and transcriptional signature consistently shows Obs/Exp <= 1*

*2) The overlap between transcriptional signature and mutated gene expression consistently shows Obs/Exp <= 1*

*3) The fraction of mutational signature or transcriptional signature in the advanced phenotype (LC metastasis: Metastatis group, LC drug response: PD, CML drug response: poor) hold steady and the fraction of mutational signature is higher than the fraction of transcriptional signature.*

*4) The overlap between evolutionary path and clonal selection analysis consistently shows Obs/Exp <= 1*

*Figure S9. Robustness of the analysis (A) The overlap of features between mutational analysis and transcriptional analysis in the evolutionary path and clonal selection analyses with different q-value thresholds (related to Figure 5D). (B) The overlap of features between transcriptional analysis from the “metastasis” or “PD” group and mutated gene expression analysis in the evolutionary path and clonal selection analyses with different q-value thresholds (related to Figure 5E). (C)The fraction of features from “metastasis,” “PD,” or “poor response” in mutational signature and transcriptional signature with different q-value thresholds (related to Figure 5F). (D) The overlap of features in the same enrichment group (mutational signature, transcriptional signature, and mutated gene expression) between the evolution path analysis and clonal selection analysis with different q-value thresholds (related to Figure 5G).*

4. Another limitation of this study and Canvolution that should be discussed is the relative scarcity of full-length single-cell RNA-seq datasets compared to the abundance of 3' end or 5' end single-cell RNA-seq datasets (such as those from 10X Genomics).

*Response: Thank you for the comment. We agree that this is a key limitation and we have revised the discussion:*

*“”” One reason for the small scope of our study is the scarcity of full-length scRNA-seq currently available in the public domain. The vast majority of scRNA-seq datasets were generated by 3’ end or 5’ end sequencing using the 10X Genomics platform. Unfortunately, SNV calling is much more challenging with this technology, and consequently it is hard to perform co-evolution analysis of genomic and transcriptomic with Canvolution. However, long-read sequencing technologies from PacBio[76] and Oxford Nanopore have recently been combined with singlecell DNA/RNA [77,78], and we anticipate that it will be straightforward to apply Canvolution to this type of data. “””*

Reviewer #2:

In this study, Cho and Hemberg introduce a new tool, Canvoltion, that is able to integrate cancer evolution and clonal selection analyses alongside transcriptional profiling using full-length single-cell RNA-seq data. The workflow joins together multiple pieces of software for mutation calling, clonal inference and tree reconstruction, gene signature enrichment and ligand-receptor interaction inference. The power of the method is highlighted on two publicly available datasets from lung cancer and chronic myeloid leukemia with available treatment response annotation. The authors generally find high clonal and transcriptional diversity linked to drug resistance, which leads them to posit that clonal sweeps are unlikely to be common during the evolution of these cancers. This original approach at inferring joint mutational and transcriptional evolution in single cell data illustrates how information from single cells can be maximised to understand cancer evolution, and can have many valuable applications in the field. The authors make the code freely available and a good tutorial is provided for using the tool on Github. The statistics employed in the study are appropriate, although not all of the metrics illustrated in the figures are entirely intuitive and could use more explanations in the figure captions. I have the following comments/suggestions that I believe would improve the clarity and robustness of the study:

1. A more detailed description of the CML and LC datasets employed in the study would be helpful - e.g. how many good and poor responders are in each category?

*Response: We apologize for the poor description of the datasets. We have revised as below:*

*“”” There were nine PD, two SD, 27 PR, and two CR; 28 of the samples were metastatic and 14 were from primary cancers. Fifteen of the donors were good responders and fourteen had a poor response. For both LC and CML information about the treatment response was not available for all samples, and some of the patients contributed more than one sample.*

*“””*

The authors highlight lack of significant differences between good and poor responders in several cases, but are they powered to identify such significant differences (assuming transcriptional signatures and clonal profiles are summarised per patient rather than per cell).

*Response: We tried to obtain our result at a sample level. For evolutionary path analysis, we performed a correlation analysis between the signature score and the depth of all the branches in each sample. For clonal selection analysis, we performed a correlation analysis between the signature score and the relative abundance of clone in each sample. However, due to the small number of samples we did not obtain any significant results (q value < 0.1).*

2. A higher clonal diversity was observed in donors with poor response to treatment in CML samples, but not LC samples. How can this discrepancy be explained?

*Response: Thank you for the comment. We did not realize this discrepancy during the first submission. We revised the discussion section below.*

*“”” Interestingly, CML samples show a higher clonal diversity for poor responders, while LC samples do not. We speculate that the difference could be due to CML being a liquid cancer while LC is a solid cancer. The two types of malignancies differ in terms of their interaction with the TME, and this may be reflected by the treatment response. “””*

3. "There were no significant differences in transcriptional diversity between the different conditions, arguing against the hypothesis that higher fitness of a tumor is associated with a more diverse mix of transcriptional states." - This is a bit of a strong statement, considering that pre-defined and fairly generic transcriptional signatures are employed. What if the signatures that are truly variable have been missed in this analysis? If the authors were to conduct an unbiased transcriptional analysis (e.g. using differential expression methods or similar), would there be no transcriptional signals arising as distinct between conditions?

*Response: Thank you for the comment. We believe that our claim is true as our analysis does not indicate any increase in the diversity of transcriptional states. However, the reviewer raises a related, but slightly different point regarding expression of specific transcriptional programs. Indeed, the reviewer is correct as there are several key genes that are different between the two conditions. In the LC dataset, metastasis-related genes (ex: MET, RAC1, CD24) and EMTrelated genes (ex: FBLN2, SDC1, CTSB) were highly expressed in metastatic cancer. Also, metastasis-related genes (ex: EPCAM, MET, RAC1, CD24) and quiescence-related genes (ex: ALOX5, CXCL5, VNN1) were highly expressed in PD patients. From the CML dataset, the DNA_damage gene (ex: RBL2) was highly expressed in poor responders. These results are consistent with our other Canvolution results and we have incorporated the analysis in the results section.*

*Taken together, these analyses show that rather than the diversity of transcriptional states found in the tumor, it is the existence of a certain transcriptional state that is important for adapting new perturbation in the tumor.*

4. Figures 5D-E: it is not clear to me what these two figures are showing, in particular what is represented on the x axis. Please detail abbreviations in the figure captions, and if possible make the labels more explicit (please do this for all figure panels where labels are cryptic). Can the authors explain this analysis in a bit more detail as well?

*Response: Sorry for the poor description in Figures 5D and E. We revised it accordingly and add more explanation below.*

*“”” Next, we tried to understand the coherence of the changes in the mutational and transcriptional profiles. When we compared the signatures that exhibited both mutations and altered expression levels, it was smaller than expected by chance for all three comparisons between evolutionary contexts (Fig. 5D). For example, even though patients from the LC cohort with PD showed an increased number of mutations in driver genes or angiogenesis mutations for both the evolutionary path and clonal selection analysis, these genes did not show increased expression levels. This result suggests that in primary cancers, genes that are mutated do not also have changes in gene expression. By contrast, the metastasis or drug-resistant groups were more likely to express a mutated gene if those features were enriched in the transcriptional evolution analysis (Fig. 5E). This implies that for more advanced cancer, many of those pathways are expressing the mutated form, and if the mutation resulted in a gain or loss of function then the function may differ from the annotated one.*

*“””*

*Also, we revised all the main figures if the labels were cryptic and minimized the white space (from comment 8).*

5. How generalisable are the findings in these two cancers to other types of cancer? While some similarities were pointed out, there were also some differences between CML and LC, so it is not entirely clear to what extent these findings are tissue and treatment specific.

*Response: Thank you for the comment. We were trying to show Canvolution can work both solid and liquid cancers. So, some discrepancies might arise from different cancer types, which is described in the response of Comment 2. On the other hand, patients from both datasets received TKI-targeted therapy. Still, there might be different usage of drugs for TKI, and some additional treatments might exist which will lead to further discrepancy. Nonetheless, given the limited number of cancer types, conditions and patients one should be cautious about generalizing the findings presented here. We mention this limitation in the limitation section:*

*“”” The fact that we were only able to study two types of cancer, with a limited number of patients and conditions means that one must be cautious about generalizing our findings to other contexts and cancer types. “””*

6. Linked to the point above, I am not sure the following statement in the abstract can generalised to other cancers "This suggests that selection based solely on growth rate is unlikely to be a major driving force during cancer evolution.", and I am also not convinced the authors have provided sufficient evidence for clonal growth not being a major driver, considering the limitations of single cell sequencing experiments analysed (e.g. only surviving cells are sampled during/after treatment, but lack of lineage tracing means a change in phenotype from one time point to the other cannot be captured; lack of spatial resolution etc.).

*Response: Thank you for the comment. We believe that our analyses support the conclusion that growth rate is not the sole driving factor. We say this based on the fact that the correlation between clonal abundance and clonal tree depth is moderate (LC: 0.5020964, CML: 0.3238787), suggesting that other factors play a role. To further explore this issue we adopted “Expanding clonal analysis” from a lineage-tracing mouse model for cancer evolution research (Dian et al, Cell, 2022). However, we could not identify any expanding clones from the default thresholds. This might be due to the less comprehensive phylogeny tree by scRNA-seq compared to the lineage-tracing model.*

*The statement that the reviewer referred to in the abstract is preceded by a sentence which states that it applies to LC and CML. Hence, we did not mean for this to be a general conclusion, so we have updated the text to make sure that this is no longer the case.*

*We have also elaborated on the additional analysis in the results on p 6.*

*“”” To investigate if more advanced clones tend to be larger, we calculated the Spearman correlation between the clonal abundance and the height in the clonal tree, and this showed moderate relation (LC: 0.5, CML: 0.32). Hence, there is only modest evidence to support the hypothesis that more advanced clones have strong growth advantage over their predecessors. “””*

7. The findings hinge on the assumption that clonal reconstruction from single cell RNA-seq data is accurate enough. The authors acknowledge the limitations in calling mutations from single cell RNA-seq data, but it would be helpful to have a sense of how the metrics would look like if mutations were called entirely randomly. Would the reconstructed trees be significantly different? How much of a dependency is there between the clonal reconstruction from inferred mutation data and the transcriptional signatures analysed?

*Response: Thank you for the comment and the suggested control. To generate the random distribution of mutation, we assigned mutation for each locus randomly. But, since we want to know how the random mutation affects the trees, we constrained the number of mutated loci to be the same as in the original data and we also retained the clonal annotation.*

*After performing transcriptional signature analysis, we obtained the mean spearman correlation (SCC) of transcriptional signatures from each sample. Since not all the signatures are represented to each sample (cancer), we only compared the absolute (mean SCC) > 0.4 between random and real data. As a result, those correlated signatures were diluted during randomization.*

8. The general aesthetics of the figures need improvement - spell out fully the axis labels without abbreviations, minimize white space etc.

*Response: We revised the figures accordingly from the reviewer’s comment 4.*

**Second round of review**

**Reviewer 1**

In this revision, the authors have appropriately addressed all of my major concerns. However, there are two minor suggestions for further improvement before publication:

1. The manuscript currently does not meet the journal's standards regarding figure visualization quality. It is important to maintain a consistent and sufficiently large font size for easy readability. Additionally, make sure that the first word in each figure caption is capitalized. Ensure a consistent visual plot style, whether it's box or bar plots, across all panels. Any undefined text abbreviations in the figures should be clarified. Moreover, consider revising the figure legends to provide a more comprehensive understanding of the figure panels.

2. The Methods section requires further elaboration to offer readers enough details for accurate replication of the analyses in the future. Include specific versions, parameters, cutoffs, and filters used for all bioinformatics tools applied in the study. Instead of solely citing the studies or papers for existing datasets used in this study, disclose the exact resources and accession numbers, where applicable.

**Reviewer 2**

The authors have addressed all my comments and the manuscript has improved substantially. Congratulations to the authors for this nice study.

**Authors Response**

**Point-by-point responses to the reviewers’ comments:**

Reviewer #1: In this revision, the authors have appropriately addressed all of my major concerns. However, there are two minor suggestions for further improvement before publication:
 1. The manuscript currently does not meet the journal's standards regarding figure visualization quality. It is important to maintain a consistent and sufficiently large font size for easy readability. Additionally, make sure that the first word in each figure caption is capitalized. Ensure a consistent visual plot style, whether it's box or bar plots, across all panels. Any undefined text abbreviations in the figures should be clarified. Moreover, consider revising the figure legends to provide a more comprehensive understanding of the figure panels.
 2. The Methods section requires further elaboration to offer readers enough details for accurate replication of the analyses in the future. Include specific versions, parameters, cutoffs, and filters used for all bioinformatics tools applied in the study. Instead of solely citing the studies or papers for existing datasets used in this study, disclose the exact resources and accession numbers, where applicable.

*Response: 1) We have improved the figures as recommended (abbreviation, capitalization, and consistency).*

*2) We have revised captions for figure 5D,E,G. We think the rest of the captions were fine.*

*3) We added all the missing versions or parameters in the methods section.*

*CTAT (v2.0.1), bcftools (v1.11), DENDRO (v0.2.2), RobustClone (no version), Seurat (v4.1.0), CellChat (v1.6.1), and oncoNEM (v1.0)*

*And other parameter settings (not shown here).*

*4) Scripts for reproducing the result from the raw fastq file are provided in both GitHub and Zenodo.*
